# Exploiting Catabolite Repression and Stringent Response to Control Delay and Multimodality of Bioluminescence Signal by Metal Whole-Cell Biosensors: Interplay between Metal Bioavailability and Nutritional Medium Conditions

**DOI:** 10.3390/bios12050327

**Published:** 2022-05-11

**Authors:** Eva Delatour, Christophe Pagnout, Marie Zaffino, Jérôme F. L. Duval

**Affiliations:** 1 Université de Lorraine, CNRS, LIEC (Laboratoire Interdisciplinaire des Environnements Continentaux), UMR7360, Campus Bridoux, F-57070 Metz, France; eva.delatour@univ-lorraine.fr (E.D.); christophe.pagnout@univ-lorraine.fr (C.P.); marie-laure.zaffino@univ-lorraine.fr (M.Z.); 2 Université de Lorraine, CNRS, LIEC, UMR7360, F-54501 Vandoeuvre-lès-Nancy, France

**Keywords:** bioluminescence, whole-cell biosensors, metals, bioavailability, medium nutritional quality, signal dependence on time

## Abstract

The time-dependent response of metal-detecting whole-cell luminescent bacterial sensors is impacted by metal speciation/bioavailability in solution. The comprehensive understanding of such connections requires the consideration of the bacterial energy metabolism at stake and the effects of supplied food on cells’ capability to convert bioaccumulated metals into light. Accordingly, we investigated the time response (48 h assay) of PzntA-*luxCDABE Escherichia coli* Cd biosensors in media differing with respect to sources of amino acids (tryptone or Lysogeny Broth) and carbon (glucose, xylose and mixtures thereof). We show that the resulting coupling between the stringent cell response and glucose/xylose-mediated catabolite repressions lead to well-defined multimodalities and shapes of the bioluminescence signal over time. Based on a recent theory for the time–response of metal-sensing luminescent bacteria, successful theoretical reconstructions of the bioluminescence signals are reported under all Cd concentrations (0–20 nM) and nutritive conditions examined. This analysis leads to the evaluation of time-dependent cell photoactivity and qualitative information on metal speciation/bioavailability in solution. Biosensor performance and the position, shape, number, and magnitude of detected peaks are discussed in relation to the metabolic pathways operative during the successive light emission modes identified here over time. Altogether, the results clarify the contributions of metal/nutrient bio-availabilities and food quality to cell response typology.

## 1. Introduction

Due to anthropogenic activities, heavy metals are ubiquitous contaminants in aquatic environments. Given their persistence and potential toxicity towards (micro)organisms, accurate (bio)analytical tools are required to detect metallic contaminants, measure their concentration and elaborate the reliable diagnosis of pollution levels. The monitoring of heavy metals in environmental samples can be performed with, e.g., electrochemical or spectroscopic methods [1,2]. The latter, however, often call for complex sample extraction protocols, are generally costly, and remain difficult to run on a routine basis for on-site measurements [3]. Most importantly, these methods rarely discriminate between bioavailable and total metal fractions in solution, a distinction that is mandatory for the proper assessment of metal-induced toxicity on biota [4].

In the past few decades, the use of whole-cell bacterial sensors for metal detection has proven to be an elegant alternative that overcomes the limitations of traditional physicochemical metal-dosing methods. These sensors are genetically-modified bacteria that produce a measurable signal (e.g., an electrochemical current or light) after they bioaccumulate metal ions from an aqueous sample [5]. Metal sensing by bacteria exploits common cell homeostasis and resistance strategies, i.e., the MerR family of metal-triggered transcriptional regulators [6]. These regulatory elements include a stress-response promoter activated after the specific binding of a given target metal to a regulatory protein, which subsequently leads to the expression of a reporter gene (i.e., *lux* or *gfp*) fused to the promotor, and to the production of reporter proteins (luciferase and GFP) at the origin of the measured signal (bioluminescence and fluorescence, respectively) [6,7]. Due to their nature as living unicellular organisms, bacterial metal sensors directly experience the bioavailability of metal compounds as they pass through the membrane, exert their effects or are transformed by the cell. They thus provide the easiest and most transparent reduction in the complexity of detecting biological impacts. Unlike a fluorescence response, bioluminescence signals are highly sensitive due to the absence of background contribution, which is attractive for metal monitoring. In addition, the functioning of *lux*-based biosensors does not require the addition of a substrate (e.g., ATP) nor recourse to a laser source for cells to produce light [3,7,8,9].

Despite these recognized benefits, the mechanistic and quantitative interpretation of the dependence of bioluminescence signals by whole-cell metal reporters on time remains an as-yet poorly resolved issue due to the complexity of the joint physical, chemical, and biological processes that govern the partitioning of metal ions at the biosensor/solution interface and the ensuing cell response. Difficulties include the proper evaluation of the interplay between the (i) bioavailability features of the metal species in the extracellular medium [10,11,12], (ii) metal biouptake flux at the biosensor surface, and (iii) kinetics of the intracellular biochemical reactions leading to photons emission [13,14]. In particular, items (i) and (ii) depend on the dynamics of metal ion speciation defined by the pertaining rate parameters for the formation and dissociation of metal complexes involving ionic, molecular and/or particulate metal ligands dispersed in solution [12,15,16]. In turn, these rate constants, together with the diffusion properties of the various metal forms, determine the lability of the complexed metal species, i.e., their contribution to the dynamic supply of free metals to the biosensor surface [12,15]. Though the Biotic Ligand Model (BLM) [17,18,19,20] and related thermodynamic metal speciation computations (e.g., Visual-MINTEQ program [21]) are commonly adopted to link empirically bioluminescence and the concentration of bioavailable metal ions (a priori assimilated by BLM to the bulk concentration of free, non-complexed metals) [22,23,24,25,26], their failure to predict metal bioaccumulation has long been reported for a variety of organisms under both metal complexing and non-complexing medium conditions ([17,27] and references in [28]). The limits of BLMs include [17,28] the restrictive and often oversimplified equilibrium-based assessment of the necessarily dynamic processes that control the reactive transfer of metal species to the surface of metal-accumulating cells and the coupling with metal biouptake and bioaccumulation [15].

Bioluminescence signals are not only impacted by the aforementioned metal speciation-related factors but also intimately dictated by the expression level of the reporter gene set under the transcriptional control of the metal-inducible promotor, and by the kinetics of reporter protein production (i.e., luciferase) [13,29,30]. These processes are themselves mediated by the physiology of the cell sensors [13,31,32] that relates to the concentration and quality of nutrients in the medium, ‘quality’ being understood here in terms of the amount of energy provided to the sensing cell and expressed per mole of nutritive compound. These nutritional conditions are obviously critical because they contribute to the maintenance of cell viability, required metabolic function, resistance function, and renewal cell functions [3]. They further condition the performance of the biosensors by supplying the energy source (e.g., amino acids and sugars) necessary to sustain the production of bioluminescence [32,33,34,35,36]. This energy demand can be more or less satisfied depending on nutrient bioavailability, cell concentration and the occurrence of nutrient complexation in solution or their adsorption onto abiotic particles [37].

Though there have been many attempts to correlate metal bioavailability and bioluminescence by whole-cell metal bioreporters from equilibrium BLM-like modelling and the use of luminescence calibration curves [22,23,24,25,26,38], very few studies have addressed how the bioavailability and quality of nutrients impact the time–response of the biosensor. Such an analysis, however, is required to ensure the applicability of the tacitly supposed equivalence between measured sample and calibration media in terms of the energy supply to the bacteria, and thereby control that biosensor performance in the former can be properly inferred from that derived in the latter. In support of this requirement, Nealson et al. [39] showed that the synthesis and activity of luciferase are connected to the metabolic state of the bacteria—more exactly to the nature of the carbon source present in the medium and to the efficiency by which cells exploit this resource. The underlying catabolite repression of bacterial luminescence therefore has direct functional implications, and luciferase cannot be considered as a nonfunctional enzyme system. Following this pioneering work, Shimada et al. [33] showed that the real-time dynamics of metabolism in *Escherichia coli* constitutively expressing *luxCDABE* could be measured by monitoring the time-dependent expression of bacterial luciferase activity on the premise that such activity is proportional to the produced bioluminescence.

Recently, Duval and Pagnout [14,32] demonstrated that the bioluminescence generated by metal-sensing *luxCDABE E. coli* is not linearly related to the cell photoactivity that is itself defined by the overall capacity of the cell sensor to produce light (which includes luciferase activity) under given nutritive conditions. Instead, they demonstrated that the bioluminescence produced at any time *t* is connected to the corresponding cell photoactivity by a convolution product that involves terms pertaining to the dynamics of bioavailable metal transfer from solution to the biosensor surface and to the kinetics of photons emission after metal internalization. They further showed that an increase in the concentration of amino acids in solution (at constant total cell concentration over time) results in a transition from mono- to bi-modal bioluminescence emission due to a global regulatory mechanism called the stringent response. Applications of the theoretical formalism elaborated by Duval and Pagnout [14] then allowed to distinguish between signal contributions associated with the flux of metal biouptake and changes in cell photoactivity following variations in amino acid bioavailability over time. For a signal controlled by cell stringent response, the initial biouptake of amino acids available in solution leads to a short-term bioluminescence peak followed by a long-term emission. The latter is associated with the use of amino acids that are self-synthesized by the cells from intermediates in glycolysis (Embden–Meyerhof–Parnas pathway, or EMP), pentose phosphate pathway (PPP) and citric acid cycle (TCA cycle) in response to significant amino acid depletion from solution generated during the short-term emission. In turn, time-dependent bioluminescence signals may be viewed as spectra reflecting all cell energy demands during the bioassay and their functional metabolic pathways in the same way as, e.g., Raman spectra discriminate chemical compounds according to their vibrational properties [32]. The possibility to fine-tune bioluminescence emission patterns over time through rational modulations of nutrient bioavailability in solution is obviously very attractive, not only for a comprehensive understanding of the intricate relationships between bioluminescence signals and metal bioavailability but also from the perspective of monitoring the toxicity of exogenous elements towards bacteria featuring different energetic trade-offs over the course of time.

In view of the above elements and following our previous work [32], the main objective of the current study is to explore how coupled stringent response and catabolite repression affect the bioluminescence response of metal-detecting *E. coli* sensors containing the *luxCDABE* operon set under the control of a Cd-inducible pZntA promotor. Briefly, depending on the extent of this coupling, adjusted here by varying concentrations in amino acids (and source thereof) in glucose and xylose, it is shown that the modality and emission delay of a bioluminescence signal can be finely tuned on demand. In detail, we demonstrate that the signal takes the form of a well-defined mono, bi, tri- or even quadri-modal emission, the magnitude of each successive peak being linearly related to the total Cd concentration in solution taken here in the 0–20 nM range where metal-induced toxicity is excluded [13,14,32]. The amplitude, time-position and shape of the observed sequential peaks are further successfully reconstructed on the basis of our recent theory [14] on kinetics of bioluminescence production by whole-cell metal *lux*-bioreporters. Ultimately, the results discussed in this work advocate for considering *time-dependent cell photoactivity* as a proxy for probing the effects of chemical stressors (e.g., lack of essential nutrients in solution) on luminescent bacterial sentinels and their metabolism in, e.g., complex environmental media. This work further evidences the benefits of measuring bioluminescence signals with min-data acquisition frequency over several tens of hours, which contrasts with current biosensing practices that are mostly restricted to the mere consideration of the only maximal signal amplitude without the mechanistic exploitation of the full cell signal dependence on time.

## 2. Materials and Methods

### 2.1. Luminescent Whole-Cell Bacterial Reporters

The bioluminescent cadmium-detecting cells adopted in this work were constructed from the BW25113 *Escherichia coli* strain obtained from the Coli Genetic Stock Center at Yale University [40]. Briefly, this biosensor was constructed by introducing the plasmid pUCD615 pZnt-lux [41] containing the *luxCDABE* operon under the control of the cadmium-inducible pZntA promoter.

### 2.2. Cell Growth Condition, and Media Composition Adopted for the Bioluminescence Assays

The cryo-preserved (−80 °C) bacterial cells were inoculated on LB-agar agar supplemented with 50 µg/mL of ampicillin and 30 µg/mL of kanamycin, and they were incubated at 37 °C for 24 h. Isolated colonies were transferred to a 125 mL Erlenmeyer flasks containing 20 mL of an LB medium (10 g/L of casein peptone, 5 g/L of yeast extract and 10 g/L of NaCl, Fisher Scientific™, Hampton, NH, USA) supplemented with 20 µL of ampicillin (50 mg/mL) and 60 µL of kanamycin (10 mg/mL), and samples were then incubated at 37 °C for 9 h under agitation at 160 rpm. Pre-cultured cells were used to inoculate at a 1:100 dilution 100 mL of an LB medium with 300 μL of kanamycin (10 mg/mL) and 100 µL of ampicillin, after which cells were incubated overnight at 37 °C under agitation at 160 rpm. The OD_600nm_ of the prepared bacterial suspensions was measured, and samples were then subdivided into 16 aliquots of 5 mL. These 16 aliquots were washed twice by centrifugation (7000× *g*, 3 min) with an nGGM medium (40 mM MOPS (Acros organics, Hampton, NH, USA), 1 mM MgCl_2_ (Sigma-Aldrich, St Louis, MO, USA), 12.5 mM NH_4_NO_3_ (Merck, Kenilworth, NJ, USA), 10 mM KNO_3_ (Normapur^®^, Radnor, PA, USA), 5 mM K_2_SO_4_ (Normapur^®^), 0.068 mM CaCl_2_ (Prolabo, Radnor, PA, USA), and 5 mM β-glycerophosphate (Sigma, St Louis, MO, USA) at pH 6.8 adjusted by the addition of 0.1 M NaOH) supplemented with tryptone (1% *v/v*) as the amino acid source (Euromedex, Souffelweyersheim, France) and glucose and xylose (Sigma-Aldrich) at different concentration ratios (100/0, 50/50, 30/70, 20/80, 18/82, 16/84, 14/86, 12/88, 10/90, 6/94, 4/96, 2/98, and 0/100). Below, we denote as *x* the ratio between glucose concentration and the sum of glucose and xylose concentrations, the explored conditions thus corresponding to *x* = 1, 0.5, 0.3, 0.2, 0.18, 0.16, 0.14, 0.12, 0.1, 0.06, 0.04, 0.02, and 0, respectively. Cell suspensions were resuspended in these media in order to obtain a final optical density of 2.0 at 600 nm. Two additional glucose/xylose concentration ratios (100/0 (*x* = 1) and 0/100 (*x* = 0)) were further prepared without tryptone supplementation. A situation in which the medium lacked both glucose and xylose was also examined. In all cases, the 100% glucose and 100% xylose reference conditions corresponded to 0.5% (*m/v*) glucose and 0.5% (*m/v*) xylose concentrations in the medium, respectively. Bioluminescence assays were further conducted upon replacing 1% (*v/v*) tryptone by 1% and 1.5% LB.

### 2.3. Bioluminescence Measurements

Bioluminescence measurements were performed in a 96-well microplate (NuncTM, Thermo Scientific^TM^, Paris, France) with a SAFAS Xenius luminometer (SAFAS, Monaco). Wells were filled with 70 µL of Milli-Q ultrapure water, 10 µL of nGGM medium, 10 µL of bacterial culture prepared along the lines detailed in Section 2.2, and 10 µL of Cd(NO_3_)_2_ (Fluka) in order to obtain a final total concentration range of Cd(II) from 0 to 20 nM (adopted values: 0, 4, 8, 12, 16 and 20 nM). The latter corresponded to a linear response of the biosensors and to the absence of metal-induced toxicity effects [13,14,32]. In turn, the concentration of tryptone and LB adopted for the bioluminescence assays was 0.1% (*v/v*) and 0.1–0.15%, respectively, and that of the nGGM-glucose/xylose medium corresponded to a 5-fold dilution of the nGGM glucose/xylose solution prepared as detailed in Section 2.2. Luminescence was measured at 490 nm every 5 min for 48 h at 25 °C, each measurement being preceded by orbital shaking for 10 sec (3 mm amplitude at 10 Hz frequency). Control experiments were carried out at a 0 nM Cd concentration, and—unless otherwise specified (i.e., in Supplementary Figure S1)—the bioluminescence responses measured at 4, 8, 12, 16 and 20 nM Cd concentrations were systematically corrected for the 0 nM reference data by simple point-by-point subtraction. It is recalled here that the bioluminescent sensor of interest in this work responds to both Cd^2+^ and Zn^2+^ metals [24]. The aforementioned subtraction eliminated the low-signal contribution of trace Zn^2+^ present in the media. An illustrative example of raw bioluminescence signals measured at 0, 4, 8, 12, 16 and 20 nM is provided in Supplementary Figure S1. We verified that the contribution of the reference signal at 0 nM Cd concentration to signals measured in the presence of Cd was insignificant under all tested medium conditions except in the long-term emission mode corresponding to xylose metabolism (i.e., in the time domain where bioluminescence in that mode decreased to 0; see Discussion). In addition, we verified that the optical density at 600 nm remained constant at a value of 0.2 for the full duration of the bioluminescence assays, which ensured that total cell concentration did not vary with time. This condition facilitated the quantitative evaluation of cell photoactivity with time and its dependence on nutritive medium conditions. The connection between cell photoactivity and bioluminescence at time *t is* briefly outlined in the next section.

## 3. Bioluminescence Response of Metal Whole-Cell Biosensors: Interpretative Framework

### 3.1. Theoretical Expression for the Bioluminescence

For medium compositions leading to a response of metal (M)-detecting whole-cell bacterial sensors featuring multiple bioluminescence peaks over time, a straightforward extension of our theory applied previously to interpret bimodal signals [14,32] leads to the following expression for the bioluminescence emitted at time *t*, denoted hereafter as ℒum(t)

(1) $$ℒum\left(t\right)=\sum_{i=1}^{m}\Psi_{i}c_{p,i}^{\max}J_{u,i}\left\{F\left(t\right)⊗{\bar{c}}_{p,i}\left(t\right)\right\}$$

where the symbol ⊗ stands for the convolution product in the time domain and *m* refers to the number of emission modes detectable over time via the corresponding *m* successive bioluminescence peaks. $\Psi_{i=1,\ldots ,m}$ (in counts s^−1^ mol^−1^ m^5^) is defined by

(2) $$\Psi_{i}=S_{a}V_{T}k_{\nu ,i}k_{f,i}\tau_{q}K_{\mathrm{Hi}}^{-1}$$

where $S_{a}$ refers to the surface area of an individual biosensor, $k_{\nu ,i}$ (counts s^−1^ mol^−1^) is the kinetic constant for photon emission per mole of luciferase in emission mode *i*, $k_{f,i}$ (mol m^−3^ s^−1^) is the kinetic constant for luciferase production in mode *i*, $\tau_{q}$ (s) is the characteristic timescale over which bioluminescence is emitted by a given luciferase–luciferin complex emitter [14,29,32], and $K_{\mathrm{Hi}}$ (mol m^−3^) is the Hill constant that is related to the affinity of the promoter-controlling *lux*-reporter gene transcription for the M–P_reg_ complex formed between M and intracellular regulatory protein P_reg_ (P_reg_ ≡ ZntR for the system of interest here). The number concentration of photoactive cells at time *t* in emission mode *i* is denoted as $c_{p,i}\left(t\right)=N_{i}\left(t\right)/V_{T}$ (m^−3^) where $V_{T}$ is the solution volume and $N_{i}\left(t\right)$ is the corresponding number of light-emitting cells. As argued elsewhere [14,30], $c_{p,i}\left(t\right)$ differs from the total concentration of cells that is constant here over the whole duration of the bioluminescence assays under the conditions of interest in this work. The dimensionless form of $c_{p,i}\left(t\right)$, hereafter denoted as ${\bar{c}}_{p,i}\left(t\right)$, is defined by ${\bar{c}}_{p,i}\left(t\right)=c_{p,i}\left(t\right)/c_{p,i}^{\max}$ where $c_{p,i}^{\max}$ (m^−3^) is the maximum concentration in photoactive cells the medium can sustain in light emission mode *i*. With this definition, only a (time-dependent) fraction of cells is photoactive and light production is viewed as an ‘on/off’ process. The quantity ${\bar{c}}_{p,i}\left(t\right)$ obviously relates to the overall efficiency of the transcription-translation processes leading to light emission at time *t*, from the metal-mediated activation of the promoter to the very production of luciferase. ${\bar{c}}_{p,i}\left(t\right)$ thus intrinsically depends on the nutritive conditions fixed by the composition of the medium [32]. In line with the arguments set forth by Delle Side et al. [30], ${\bar{c}}_{p,i}\left(t\right)$ in Equation (1) can be equivalently interpreted as a dimensionless, time-dependent function—hereafter termed cell photoactivity in emission mode *i*—that modulates the bioluminescence yield of all cells between 0 (situation at *t* = 0) and 1 (situation met at sufficiently large *t*), with light emission thus proceeding according to a continuous process over time.

The (dimensionless) function F(t) involved in Equation (1) defines the bioluminescence response at *t* to the hypothetical pulse of cell concentration $c_{p,i}\left(t\right)\equiv c_{p,i}^{\max}\delta \left(t\right)$, with δ(t) the Dirac impulsion at time *t* [14]. An analysis detailed elsewhere [14] showed that under conditions of practical interest F(t) can be recast in the form

(3) $$F\left(t\right)=\left(e^{-k_{r}t}-\frac{k_{\mathrm{eff}}}{k_{r}}e^{-k_{\mathrm{eff}}t}\right){\left(1-\frac{k_{\mathrm{eff}}}{k_{r}}\right)}^{-1}$$

where $k_{r}$ (s^−1^) is the kinetic constant associated with the reverse component of effective luciferase production reaction and $1/k_{r}$ can accordingly be viewed as a timescale for the half-life of luciferase. For media in line with insignificant metal complexation, the timescale $1/k_{\mathrm{eff}}$ (s) is defined by [14]

(4) $$1/k_{\mathrm{eff}}=\bar{K}\left(1+Bn^{-1}\right)/k_{e}$$

where $\bar{K}$ is the dimensionless stability constant of M–ZntR complexes, $k_{e}$ (in s^−1^) is the kinetic constant for M excretion and Bn is the dimensionless Bosma number (also termed the bioavailability number). This number is defined by the ratio $Bn=\left(D_{M}a^{-1}\right)/\left(k_{\mathrm{int}}K_{H}\right)$, which compares the mass transfer coefficient of metal ions by diffusion (also called the diffusion conductance)—i.e., $D_{M}\times a^{-1}$, with $D_{M}$ (m^2^ s^−1^) and a (m) being the M diffusion coefficient in solution and the cell radius, respectively—to the mass transfer coefficient associated with M internalization (or internalization conductance), i.e., $k_{\mathrm{int}}\times K_{H}$ where $k_{\mathrm{int}}$ (s^−1^) is the kinetic constant for metal internalization and $K_{H}$ (m) is the Henry coefficient for the adsorption of M on the transporter sites located at the cell membrane. For the sake of simplicity, the conductive acceleration of M diffusion in the double layer electric field at the cell/solution interface is not included in the above expression of Bn [42,43]. In the linear Henry regime of M internalization and in the absence of M depletion from bulk solution, the metal biouptake flux in mode *i*, denoted as $J_{u,i}$ (mol m^−2^ s^−1^) in Equation (1), is defined by [14,15]:

(5) $$J_{u,i}\equiv J_{u}={\left(1+Bn^{-1}\right)}^{-1}k_{\mathrm{int}}K_{H}c_{M,f}^{*}$$

where $c_{M,f}^{*}$ is the free metal concentration in bulk solution; here, we have ignored the terms pertaining to passive metal biosorption, which is legitimate for sufficiently dilute cell suspensions [13]. In Equation (5), the ‘pristine’ internalization conductance $k_{\mathrm{int}}K_{H}$ is that when there is no limitation in the energy that is required for the active transfer of M across the membrane via dedicated protein transporters, i.e., $k_{\mathrm{int}}K_{H}$ is independent of emission mode *i*. $k_{\mathrm{int}}K_{H}$ corresponds to the ratio $J_{u}^{*}/K_{M}$, where $J_{u}^{*}$ (mol m^−2^ s^−1^) is the maximum biouptake flux reached at the full saturation of the internalization sites by M, and $K_{M}$ (mol m^−3^) is the characteristic affinity of M for these sites. Pinheiro et al. [44] extended Equation (5) for cases where metal complexation by ligands L in solution is significant (see Equation (19) in [44]) and L are in excess over M. For orientational consideration, we hereafter consider the strongest ligand L that feature the highest affinity to M. Then, the result by Pinheiro et al. [44] can be rewritten in the form

(6) $$J_{u,i}\equiv J_{u}={\left(1+{\left(pBn\right)}^{-1}\right)}^{-1}{\left(1+{\bar{K}}_{s}\right)}^{-1}k_{\mathrm{int}}K_{H}c_{M}^{*}$$

where $c_{M}^{*}$ is the total bulk concentration of metal species (i.e., the free metal forms and the complexed metal species, ML) that is related to $c_{M,f}^{*}$ according to $c_{M,f}^{*}={\left(1+{\bar{K}}_{s}\right)}^{-1}c_{M}^{*}$—with ${\bar{K}}_{s}=K_{s}c_{L}$ being the dimensionless stability constant of ML complex in solution and $c_{L}$ is the bulk L concentration (mol m^−3^). The parameter p in Equation (6) is a dimensionless quantity that formulates the bioavailability of ML, i.e., the extent of its contribution to M biouptake flux following ML diffusion to the cell surface and interconversion kinetics between ML and M. In the limit of inert complexes, the conversion of M into ML and ML into M is very slow on the timescale of M and ML diffusions from bulk solution to the bioaccumulating surface, and p=1. In the other dynamic limit of fully labile ML complexes, ML↔M chemical equilibrium is maintained all along M and ML diffusions to the cell surface because rates of association/dissociation are here fast on the M/ML diffusion timescales: p is then given by $p=\left(1+\varepsilon {\bar{K}}_{s}\right)$ [44], where ε is the ratio between ML and M diffusion coefficients. It is emphasized that the popular Biotic Ligand Model (BLM) strictly and solely applies to metals and bioaccumulating systems for which p=1 and $Bn^{-1}\to 0$, and under non-depletive bulk medium conditions [14,15,17,28,43,44]. A comparison between Equations (5) and (6) indicates that the transition from a non-complexing metal situation to a metal-complexing scenario effectively comes to replace $Bn^{-1}$ with $Bn^{-1}/p$ while accounting for the relationship between $c_{M,f}^{*}$ and $c_{M}^{*}$, i.e., $c_{M,f}^{*}={\left(1+{\bar{K}}_{s}\right)}^{-1}c_{M}^{*}$. In turn, the expression of $1/k_{\mathrm{eff}}$ given by Equation (4) for non-metal complexing cases becomes the following after correcting for dynamic metal speciation

(7) $$1/k_{\mathrm{eff}}=\bar{K}\left[1+{\left(pBn\right)}^{-1}\right]/k_{e}$$

By combining Equations (1) and (6), we can show that the maximum in bioluminescence produced in emission mode *i*, denoted as $ℒum_{\max,i}$, increases linearly with increasing $c_{M}^{*}$. The corresponding slope satisfies the expression

(8) $$\partial ℒum_{\max,i}/\partial c_{M}^{*}=\Psi_{i}c_{p,i}^{\max}k_{\mathrm{int}}K_{H}{\left(1+{\left(pBn\right)}^{-1}\right)}^{-1}{\left(1+{\bar{K}}_{s}\right)}^{-1}\left\{F\left(t_{\max,i}\right)⊗{\bar{c}}_{p,i}\left(t_{\max,i}\right)\right\}$$

where $t_{\max,i}$ is defined by $ℒum\left(t=t_{\max,i}\right)=ℒum_{\max,i}$, with $t_{\max,i}<t_{\max,i+1}$. For situations where a threshold value of $c_{M}^{*}$ is required for cells to emit light in emission mode *i*, Equation (8) remains valid upon specifying the constrain ${\bar{c}}_{p,i}\left(t\right)=0$ for $c_{M}^{*}\leq c_{M,i}^{*,\mathrm{ind}}$, with $c_{M,i}^{*,\mathrm{ind}}$ being the minimum bulk M concentration value required for bioluminescence induction in emission mode *i*. Equation (8) highlights the intricate balance between physicochemical and energetic contributions to bioluminescence, the combination of which defines the overall performance of the metal whole-cell biosensor in emission mode *i*. For example, a medium with poor metal complexation capacity (i.e., low ${\bar{K}}_{s}$) may well lead to a lower $\partial ℒum_{\max,i}/\partial c_{M}^{*}$ compared to that measured in another medium where metal complexation is significant but nutritional quality is higher (larger ${\bar{K}}_{s}$ and ${\bar{c}}_{p,i}\left(t_{\max,i}\right)$). This is the direct consequence of the interplay between metal bioavailability in solution on one hand and nutrient bioavailability/quality on the other.

### 3.2. Methodology for Quantitative Reconstruction of Measured Time-Dependent Bioluminescence Signals

The straightforward rewriting of Equation (1) leads to the following expression of ℒum(t) for the illustrative case m=3

(9) $$\frac{ℒum\left(t\right)}{ℒum_{\max,1}}=\frac{F\left(t\right)⊗{\bar{c}}_{p,\mathrm{eff}}\left(t\right)}{F\left(t_{\max,1}\right)⊗{\bar{c}}_{p,1}\left(t_{\max,1}\right)}$$

where F(t) is given by Equation (3) and ${\bar{c}}_{p,\mathrm{eff}}\left(t\right)$ is the (dimensionless) cell photoactivity defined at any time *t* by

(10) $${\bar{c}}_{p,\mathrm{eff}}\left(t\right)={\bar{c}}_{p,1}\left(t\right)+\sum_{i=2}^{3}\frac{\Psi_{i}J_{u,i}c_{p,i}^{\max}}{\Psi_{1}J_{u,1}c_{p,1}^{\max}}{\bar{c}}_{p,i}\left(t\right)$$

For the sake of generality, we have conserved in Equation (10) the *i*-indexation for the metal biouptake flux. Given their definitions in Section 3.1, ${\bar{c}}_{p,i=1,..,m}\left(t\right)$ verifies the condition ${\bar{c}}_{p,i=1,..,m}\left(t\to \infty \right)\to 1$ unless otherwise specified. They should further comply with the initial boundary ${\bar{c}}_{p,i=1,..,m}\left(t=0\right)=0$ to ensure the condition ℒum(t=0)=0. The modeling of the cell signal by Equations (9) and (10) offers the advantage of expressing ℒum(t) in a non-dimensional form and to make explicit in Equation (10) the ratios $\frac{\Psi_{i=2,3}J_{u,i=2,3}c_{p,i=2,3}^{\max}}{\Psi_{1}J_{u,1}c_{p,1}^{\max}}$ that express the gain or loss in cell photoactivity within emission modes 2 and 3 in comparison to mode 1 taken as an ‘’internal reference’’. Using Equation (9), the methodology adopted to reconstruct the measured response of the metal biosensors involves three steps: (i) for each metal concentration $c_{M}^{*}$ tested, normalize the measured ℒum(t) (after their correction for the background response collected at 0 nM Cd concentration) by the corresponding $ℒum_{\max,1}$; (ii) find the required ${\bar{c}}_{p,1}\left(t\right)$, $1/k_{r}$ and $1/k_{\mathrm{eff}}$ to reproduce the time-dependence of ℒum(t) measured in emission mode 1; (iii) find the required ${\bar{c}}_{p,i=2,3}\left(t\right)$ and the dimensionless scalars $\frac{\Psi_{i=2,3}J_{u,i=2,3}c_{p,i=2,3}^{\max}}{\Psi_{1}J_{u,1}c_{p,1}^{\max}}$ that recover the biosensor response measured over the time windows corresponding to the emission modes 2 and 3 with the constants $1/k_{r}$ and $1/k_{\mathrm{eff}}$ determined in step (ii) and used as fixed parameters for each considered $c_{M}^{*}$ condition. In turn, the completion of steps (i)–(iii) leads to the evaluation of ${\bar{c}}_{p,\mathrm{eff}}\left(t\right)$ via Equation (10). Similarly to our procedure adopted in [32], the searched functions ${\bar{c}}_{p,i}\left(t\right)$ in steps (ii)–(iii) correspond to Gompertz law commonly adopted for modelling cell growth curves or to increasing sigmoidal function of time that involves the error (erf) function (case of emission mode 1). For cases where the truncation of bioluminescence peak(s) is detected (see details in Section 4.1), the corresponding abrupt decay of bioluminescence with time is recovered upon correcting ${\bar{c}}_{p,i}\left(t\right)$ with help of a decreasing exponential function of time. Data fitting to Equations (9) and (10) was systematically performed on the basis of the Levenberg–Marquardt procedure, and the PTC Mathcad Prime code developed for that purpose is available on request. For a given measured bioluminescence signal, we systematically verified that the relative error in the estimates of ${\bar{c}}_{p,\mathrm{eff}}\left(t\right)$ (at fixed time *t*), $1/k_{r}$ and $1/k_{\mathrm{eff}}$ did not exceed 10%.

## 4. Results and Discussion

### 4.1. Impacts of Nutritive Conditions on the Modality of the Bioluminescence Signal

**Figure 1:**
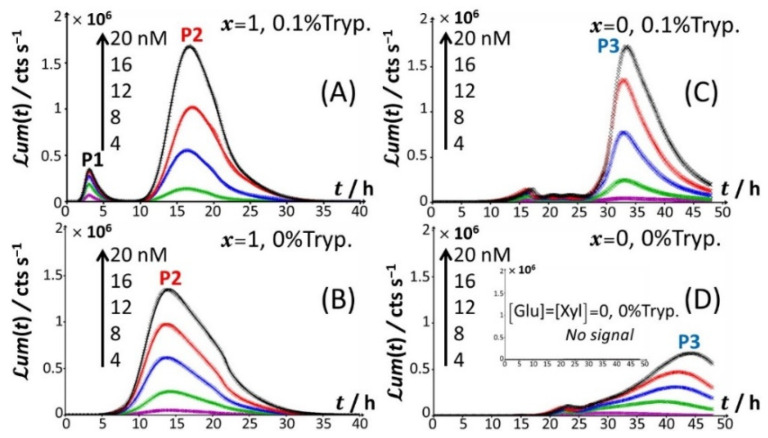
Time-dependent bioluminescence (*ℒum*(*t*) in counts per second) produced by Cd-responsive whole-cell biosensors in the presence of glucose and the absence of xylose (*x* = 1) and vice versa (*x* = 0), with or without the supplementation of 0.1% tryptone (abbreviated as Tryp.), as specified in panels (**A**–**D**). Measurements are reported for different total Cd concentrations in the range of 0–20 nM (indicated). *t* = 0 corresponds to the introduction of Cd in solution and the start of bioluminescence measurement. Signals were corrected by point-by-point subtraction of the corresponding cell response measured in 0 nM Cd concentration.

Figure 1 reports the time-dependent bioluminescence response of Cd-responsive biosensors as a function of total Cd concentration in solution in the presence and absence of amino acids (Figure 1A–D) for the nutritive conditions *x* = 1 (glucose-containing solution without xylose; Figure 1A,B) and *x* = 0 (xylose-containing solution without glucose; Figure 1C,D).

**Figure 2:**
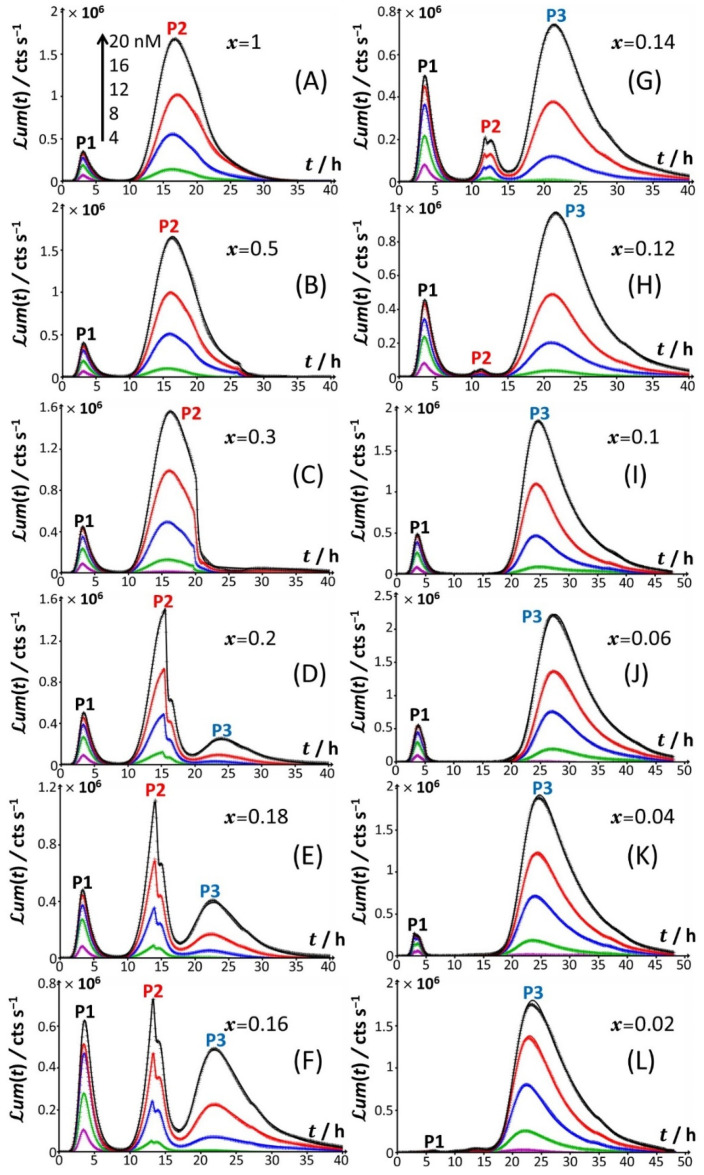
Time-dependence of bioluminescence (*ℒum*(*t*)) measured for the Cd-responsive *E. coli* biosensor as a function of total Cd concentration in the range of 0–20 nM (indicated) in nGGM media supplemented with 0.1% tryptone and different concentrations of glucose and xylose, as subsumed in *x* with 0.02 ≤ *x* ≤ 1. The scale in Cd concentration and associated color nomenclature specified in (**A**) apply to panels (**B**–**L**). (+) symbols: experimental data. Lines: reconstruction on the basis of the theory outlined in Section 3. Signals were corrected by point-by-point subtraction of the corresponding cell response measured in the 0 nM Cd concentration. The quality of theoretical reconstructions of the bioluminescence signals normalized following Equation (9) is reflected by the root mean square values reported in Supplementary Table S1.

Under all examined medium conditions, the cell response took the form of bell-shaped signal(s) with a marked dependence on Cd concentration, which validates the genetic construction of a Cd-sensing *E. coli* strain. In the presence of both amino acids and glucose (Figure 1A), the bioluminescence emission is bimodal, with the appearance of a short-term peak (P1) at *t*_max,1_~3 h and a long-term peak (P2) at *t*_max,2_~16 h, where *t*_max,1_ and *t*_max,2_ correspond to the time positions where maxima in P1 and P2 are achieved, respectively. This bimodality is qualitatively in line with the bioluminescence data reported by Duval and Pagnout [32] for another Cd-sensing luxCDABE *E. coli* strain (JW3596) measured in a diluted nGGM medium (source of glucose) supplemented with LB as the source of amino acids (instead of tryptone in Figure 1A). Briefly, the appearance of the two successive peaks originates from the stringent cell response. The first luminescence emission (peak P1) is controlled by the absorption of amino acids bioavailable in the medium and brought by tryptone. When there is a shortage of amino acids in solution, the level of uncharged tRNA critically increases, which results in the production of alarmones (p)ppGpp. These alarmones reprogram the resource-consuming processes by decreasing the levels of transcriptional machinery of tRNA and ribosome synthesis and by activating the transcription of genes for the biosynthesis of amino acids. In turn, cells recover their capacity to produce bioluminescence once they have self-palliated the deficiency of amino acids in solution, which is reflected by the appearance of the peak P2 [32,45]. In agreement with this stringent-response mechanism, the suppression of the amino acid source from the medium at *t* = 0 leads to the only peak P2 and the absence of peak P1 (Figure 1B). Under conditions where the medium contain amino acids but glucose is replaced by xylose (Figure 1C), cell response develops into a distinctive peak (hereafter denoted as P3) appearing at *t*_max,2_~33–35 h, and this peak is preceded by wavy modulations of the bioluminescence in the time range of 15–25 h. Given the times at which P1 and P2 peaks are observed in Figure 1A,B and the one marking the appearance of P3 in Figure 1C, the shift from the glucose-to-xylose carbon source obviously and significantly delays the cell response and modify the cell energetic trade-off, leading to the aforementioned stringence-controlled production of luminescence. Remarkably, starting from the situation in Figure 1C where xylose is the only carbon source, the suppression of amino acids in solution (Figure 1D) leads to vanishing of the pre-cited wavy modulations in bioluminescence and to a better-defined signal located at the foot of peak P3 that then spreads over a larger time window (*t*_max,3_~30–45 h depending on Cd concentration). Finally, the lack of both amino acid and sugar carbon sources in the medium (inset Figure 1D) result in the absence of any bioluminescence production, as expected from the recognized key importance of sugars as source of energy for bacteria. In order to refine our analysis of the way bioluminescence response is mediated by nutriment bioavailability (i.e., presence/absence of amino acids and presence/absence and nature of sugar components), the cell response patterns featured in Figure 1 for the extremes *x* = 0 and *x* = 1 are detailed in Figure 2 for conditions that cover a large range of *x*-values between 0.02 and unity in a medium containing 0.1% tryptone.

**Figure 3:**
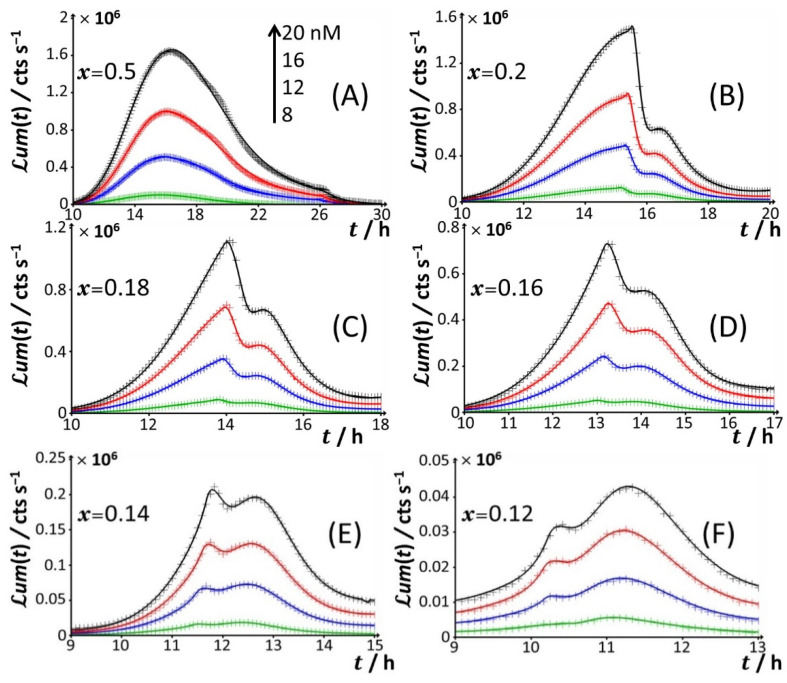
Time-dependence of bioluminescence (*ℒum*(*t*)) measured for the Cd-responsive *E. coli* biosensor as a function of total Cd concentration in the range of 0–20 nM (indicated) in nGGM media supplemented with 0.1% tryptone and different concentrations of glucose and xylose (subsumed in the variable *x*). Data are given for selected values of *x*, and they refer to the only peak P2 identified in Figure 1 and Figure 2. The scale in Cd concentration and associated color nomenclature specified in (**A**) apply to panels (**B**–**F**). (+) symbols: experimental data. Lines: reconstruction on the basis of the theory outlined in Section 3. Signals were corrected by point-by-point subtraction of the corresponding cell response measured in 0 nM Cd concentration. See text for details.

Starting from Figure 2A, which corresponds to the nutrient conditions examined in Figure 1A (*x* = 1) and to a bimodal bioluminescence emission controlled by a stringent cell response (peaks P1 and P2 at *t*_max,1_~3 h and *t*_max,2_~16 h, respectively), a decrease in glucose concentration from *x* = 1 to 0.5 and 0.3 (Figure 2B,C) results in a truncated peak P2. The truncation appears at a timepoint hereafter denoted as *t*_2_*, with t_2_*~26 h and 20 h for *x* = 0.5 and 0.3, respectively. This truncation materializes in an abrupt drop of the bioluminescence with time for *t* > t_2_* and a resulting discontinuity of the derivative of the signal with respect to time at *t*= t_2_*. In addition, the shape and time-position of peak P1 remain basically unchanged, as is the time position of peak P2 compared to the case *x* = 1. With decreasing *x* from 0.3 to 0.2 (Figure 2D), the truncation of peak P2 is shifted to a shorter timepoint (t_2_*~15 h), in agreement with the trend commented for t_2_* when decreasing *x* from 0.5 to 0.3. As a result, the time-position of the maximum of peak P2 slightly decreases to *t*_max,2_~ *t*_2_*~15 h. The new features in Figure 2D are the appearance of a right shoulder in (the descending part of) the P2 signal (i.e., for t_2_* < *t* ≤ ~20 h) and the emergence of a third signal (peak P3) that spans over the time range of ~20 h ≤ *t* ≤ ~40 h and reaches a maximum at *t*_max,3_~24 h. The amplitude of peak P3 further increases with Cd concentration in solution. A progressive decrease in *x* from 0.2 to 0.12 (Figure 2D–H) leads to an increase in the magnitude of peak P3 at a fixed Cd concentration and to a concomitant gradual extinction of peak P2 (Figure 3). This extinction is accompanied by a truncation of the peak P2 that then appears earlier with time (decreasing *t*_2_*), consistent with the trend described above for *t*_2_* and 0.5 ≤ *x* ≤ 0.2 (Figure 3). The associated discontinuity of the P2 signal, very well-observed at *x* = 0.3 (Figure 2C) and *x* = 0.2 (Figure 2D and Figure 3B), leaves place at lower values of *x* to a P2 signal that looks more like a doublet (Figure 3D–F). This transition stems from the fact that the shoulder in peak 2 (as clearly observed at, e.g., *x* = 0.2) has gradually ‘cropped’ the whole P2 signal upon decreasing *x*. In turn, this leads to a ratio between the two sequential (short-term and long-term) extrema identifiable within peak P2 that ranges from values larger than unity for 0.3 ≤ *x* ≤ 0.16 (Figure 3B–D), to ~1 for *x* = 0.14 (Figure 3E), and to values lower than unity for *x* = 0.12 (Figure 3F). Due to this increased contribution of the shoulder, positioned at the right side of the peak P2 at *x* = 0.2, the delay of the appearance of peak P2 (*t*_max,2_) is shifted to shorter times when switching the carbon source from glucose to xylose in the range of 1 ≤ *x* ≤ 0.12 (Figure 2 and Figure 3), decreasing from *t*_max,2_~16 h at *x* = 1 to *t*_max,2_~11.5 h at *x* = 0.12.

**Figure 4:**
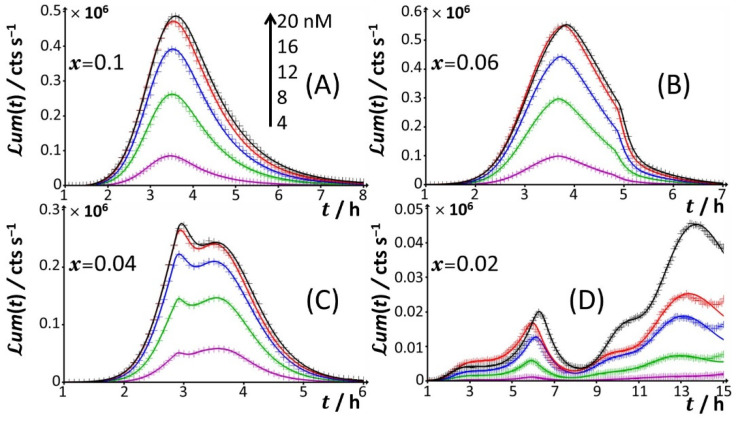
Time-dependence of bioluminescence (*ℒum*(*t*)) measured for the Cd-responsive *E. coli* biosensor as a function of total Cd concentration in the range of 0–20 nM (indicated) in nGGM media supplemented with 0.1% tryptone and different concentrations of glucose and xylose (subsumed in the variable *x*). Data are given for selected values of *x*, and they refer to the only peak P1 identified in Figure 1 and Figure 2. The scale in Cd concentration and associated color nomenclature specified in (**A**) apply to panels (**B**–**D**). (+) symbols: experimental data. Lines: reconstruction on the basis of the theory outlined in Section 3. Signals were corrected by point-by-point subtraction of the corresponding cell response measured in 0 nM Cd concentration. See text for details.

For glucose concentrations corresponding to *x* < 0.12 (Figure 2I–L), peak P2 disappears and the signal takes the form of a P1–P3 bimodal emission. Remarkably, at sufficiently low *x* in line with such a P1–P3 signal (i.e., *x* = 0.06), peak P1 is distinctly truncated (Figure 2J and Figure 4B) in a way similar to that previously detailed for peak P2 at *x* = 0.5 and *x* = 0.3. At lower values of *x* (i.e., *x* = 0.04; Figure 4C), the truncated peak P1 splits into two sub-peaks whose respective amplitudes clearly increase with Cd concentration before leveling off for 16–20 nM Cd in solution. The shape of the resulting doublet qualitatively conforms to that of peak P2 observed at *x* = 0.14 (Figure 3E). At *x* = 0.02 (Figure 2L and Figure 4D), the defining properties of the P1 doublet are no longer recognizable in the bioluminescence emission pattern. Indeed, the magnitude of P1 peak has here largely decreased, and the overall cell response is then dominated by the peak P3 preceded by the wavy modulations in bioluminescence shown in Figure 1D for glucose-lacking medium.

Remarkably, when replacing the amino acid source (0.1% tryptone) with 0.15% LB, the basic features of the cell response shown in Figure 2 when changing glucose concentration remain qualitatively similar (Supplementary Figure S2). Specifically, the biosensor signal changes from a bimodal P1–P2 signal to a P1-truncated P2 signal with gradually decreasing *x*, followed by a trimodal P1–P2–P3 emission, a bimodal P1–P3 response, and (finally) a bioluminescence response dominated by the only peak P3 at sufficiently low values of *x*. The presence of a shoulder in the severely truncated peak P2, as illustrated in Figure 2, is also shown in Figure S2 (e.g., in panel D) despite the lower frequency of bioluminescence data acquisition adopted for measurements in LB (one measurement every 15 min instead of every 5 min; see Figure 2), which makes the observation of signal truncation more difficult. Last, for a selected range of *x* conditions, we verified that changing LB concentration (Figure S3, 0.1% LB) qualitatively leads to the preservation of the different bioluminescence patterns measured in 0.15% LB. The observed differences are only relative to the respective magnitudes of the detected peaks P1, P2, and P3. This latter result echoes our previous finding on stringence-controlled cell response and the related LB concentration-dependent ratio between the maxima of peaks P1 and P2 [32].

Prior to detailed discussion of the mechanisms underlying the remarkable bioluminescence time-profiles with changing concentrations in amino acids and sugars (Section 4.3), we quantitatively analyze the way the maxima of peaks P1, P2 and P3 depend on nutritive conditions and Cd concentration in solution.

### 4.2. Impacts of Nutritive Conditions on Bioluminescence Maxima

**Figure 5:**
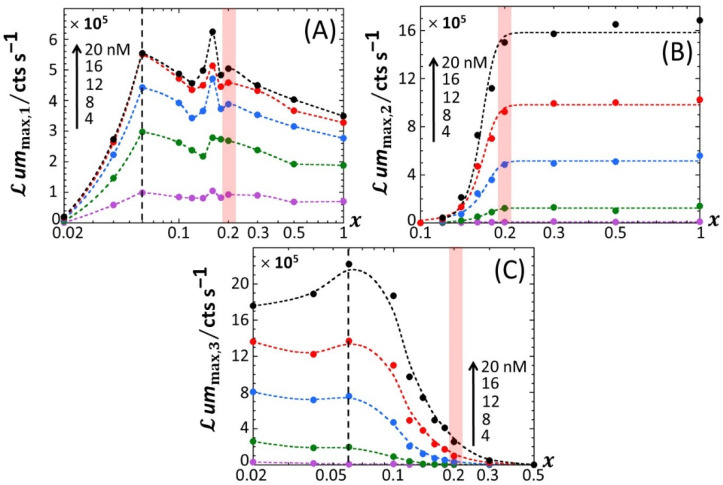
Variations of the maxima of the bioluminescence peaks P1 (**A**), P2 (**B**) and P3 (**C**) with *x* and total Cd concentration in solution (indicated). The scale in Cd concentration and associated color nomenclature specified in (**A**) apply to panels (**B**,**C**). (+) symbols: experimental data. Lines: guides for the eyes. The red-colored zone indicates the transition from P1–P2 to P1–P2–P3 signals. The vertical dotted lines correspond to the common positioning of the local maxima in ℒ*um*_max,3_ and the onset of the abrupt decrease in P1 amplitude with decreasing *x*. Measurement conditions: nGGM media supplemented with 0.1% tryptone and different concentration ratios of glucose and xylose (subsumed in the variable *x*). Data in this figure originate from those given in Figure 2 (corrected for the reference at 0 nM Cd concentration).

**Figure 6:**
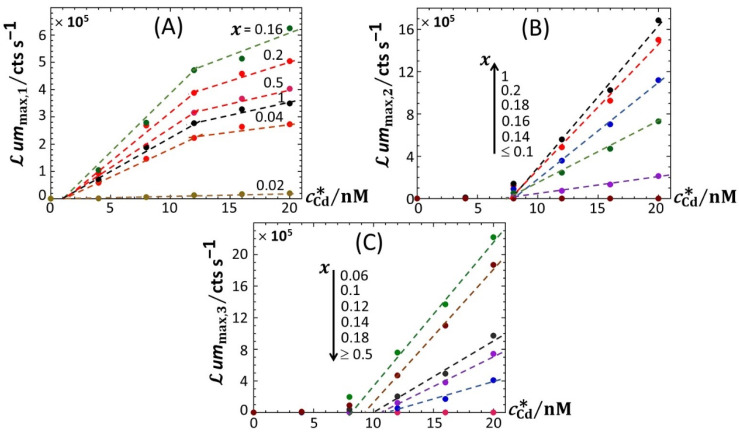
Linear dependence of the maxima of the bioluminescence peaks P1 (**A**), P2 (**B**) and P3 (**C**) on total Cd concentration in solution (indicated) at selected values of *x* (indicated). (+) symbols: experimental data. Lines: linear regressions. Measurement conditions: nGGM media supplemented with 0.1% tryptone and different concentrations of glucose and xylose (subsumed in the variable *x*). Data in this figure originate from those given in Figure 2 (corrected for the corresponding reference measured at 0 nM Cd concentration).

Figure 5 reports the dependence of the maxima of the peaks P1, P2 and P3—denoted as *ℒum*_max,1_, *ℒum*_max,2_ and *ℒum*_max,3_, respectively—on the dimensionless glucose to total sugar concentration ratio, *x* and on the total Cd concentration, $c_{\mathrm{Cd}}^{*}$, under the conditions detailed in Figure 2, Figure 3 and Figure 4 (0.1% tryptone). The critical value of *x* where peak P3 appears in the biosensor response, denoted hereafter as x* with x*~0.2, is explicitly indicated by the red-colored transition zone in Figure 5. In the range of *x*-values satisfying *x* > x*, the biosensor response displays a P1–P2 bimodality over time with *ℒum*_max,1_ that somewhat increases with decreasing *x* from 1 to x* for $c_{\mathrm{Cd}}^{*}$ ≥ 8 nM (Figure 5A). In addition, at fixed *x* *ℒum*_max,1_ clearly levels off for $c_{\mathrm{Cd}}^{*}$ ≥ 16 nM. In contrast, under such conditions, *ℒum*_max,2_ remains independent of *x* and increases with $c_{\mathrm{Cd}}^{*}$ in the whole range of examined metal concentration (Figure 5B). With decreasing glucose concentration from *x* = x* to 0.02, *ℒum*_max,2_ monotonously decreases to 0 regardless of Cd content in solution. This suppression of peak P2 is combined with the appearance of peak P3, whose magnitude *ℒum*_max,3_ increases with decreasing *x* from *x* = x* to 0.02 while passing through a maximum (at *x*~0.06) that is most pronounced at large $c_{\mathrm{Cd}}^{*}$ (Figure 5C). This transition from a P1–P2 to P1–P3 bimodal emission is further clearly marked by a local maximum in *ℒum*_max,1_ at *x* = 0.16 (Figure 5A), which is followed by a further increase in *ℒum*_max,1_ with decreasing *x* from 0.16 to 0.06 and by a massive decrease in *ℒum*_max,1_ with decreasing *x* from 0.06 to 0.02. Remarkably, the position *x*~0.06 of the maximum in *ℒum*_max,3_ corresponds to the onset of the significant decrease in *ℒum*_max,1_ with decreasing *x* (see vertical dotted lines in Figure 5A,C). Figure 6A–C highlights how the increase in *ℒum*_max,1_, *ℒum*_max,2_ and *ℒum*_max,3_ with increasing $c_{\mathrm{Cd}}^{*}$, respectively, is impacted by switching the carbon source from glucose to xylose. Starting with Figure 6B (Figure 6C), *ℒum*_max,2_ (*ℒum*_max,3_, respectively) increases linearly with $c_{\mathrm{Cd}}^{*}$, and the more so as *x* increases (decreases, respectively), in agreement with the qualitative inspections of Figure 5B,C. The data further indicate that the threshold value of Cd concentration above which bioluminescence is produced in the emission mode corresponding to peak P2 (ca. 8 nM) remains independent of *x* but decreases for peak P3 from ~11 to ~8 nM with decreasing *x* from 0.18 to 0.06. More remarkable are the data pertaining to peak P1 (Figure 6A). Indeed, putting aside the *x*-conditions corresponding to a significant extinction of peak P1 (i.e., *x* < 0.04; see Figure 5A), for this emission mode, we note that the rate of linear increase in *ℒum*_max,1_ with increasing $c_{\mathrm{Cd}}^{*}$ is clearly a function of *x* for $c_{\mathrm{Cd}}^{*}$ < 12 nM, whereas this dependence is less marked at larger Cd concentrations. In turn, this feature marks a change in the slope of the plot *ℒum*_max,1_ versus $c_{\mathrm{Cd}}^{*}$ at $c_{\mathrm{Cd}}^{*}$ = 12 nM.

**Figure 7:**
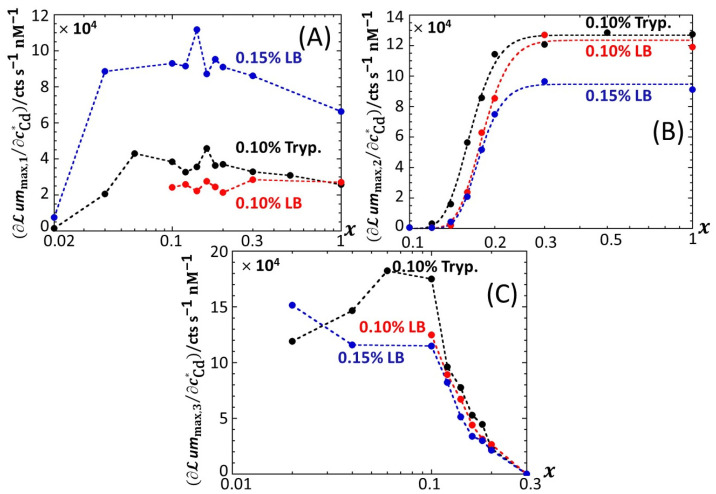
Slopes associated with the linear regressions of *ℒum*_max,1_ (**A**), *ℒum*_max,2_ (**B**) and *ℒum*_max,3_ (**C**) versus $c_{\mathrm{Cd}}^{*}$ for the various *x*-conditions tested in 0.1% tryptone, 0.1% LB and 0.15% LB media (specified). (+) symbols: experimental data. Lines: guides for the eyes. Data in this figure originate from those given in Figure 2, Figures S2 and S3 (corrected for the corresponding reference measured at 0 nM Cd concentration).

For the sake of comparison, we report in Figures S4 and S5 the equivalent of Figure 5 for 0.1% and 0.15% LB media, respectively, containing glucose-to-xylose concentration ratios in the range of 0.1 ≤ *x* ≤ 1 and 0.02 ≤ *x* ≤ 1, respectively. In agreement with Figure 2, Figures S2 and S3, the findings revealed by Figure 5 for *ℒum*_max,1_, *ℒum*_max,2_ and *ℒum*_max,3_ in 0.1% tryptone medium also apply in 0.1% and 0.15% LB media. In particular, the presence of local maxima in the variation of *ℒum*_max,1_ with *x* is confirmed (Figure 5A, Figures S4A and S5A) in the range of 0.1 < *x* < 0.2, especially at large $c_{\mathrm{Cd}}^{*}$. Noticeably, the decrease in *ℒum*_max,3_ measured at $c_{\mathrm{Cd}}^{*}$ = 20 nM when decreasing *x* from 0.06 to 0.02 in 0.1% tryptone (Figure 5C) is no longer observed in 0.15% LB (Figure S5C). Unlike in 0.1% tryptone and 0.15% LB media, *ℒum*_max,1_ remains further constant with decreasing *x* from 1 to 0.3 in 0.1% LB over the whole range of adopted $c_{\mathrm{Cd}}^{*}$ values (Figure S4A). As the analogue of Figure 6 pertaining to data in 0.1% tryptone medium, Figure S6 evidences linear dependences of *ℒum*_max,1_, *ℒum*_max,2_ and *ℒum*_max,3_ on $c_{\mathrm{Cd}}^{*}$ in 0.1% LB with varying *x* values (the same holds for the 0.15% LB medium; data not shown). The minimum Cd concentrations for the induction of bioluminescence in the P1 and P2 emission modes (3 and 6–7 nM, respectively) are comparable to those estimated in 0.1% tryptone, whereas the minimal Cd concentration for light induction in the P3 emission mode is independent of *x* in 0.1% LB (ca. 11 nM instead of 8 nM-11 nM for 0.1% tryptone depending on *x*). Interestingly, the presence of the two Cd concentration regimes identified in Figure 6A from the change in the rate of increase in *ℒum*_max,1_ with varying $c_{\mathrm{Cd}}^{*}$ in 0.1% tryptone is also observed in 0.1% LB (Figure S6A), though the corresponding transition (positioned at $c_{\mathrm{Cd}}^{*}$= 12–15 nM, depending on *x*) in 0.1% LB is lesser marked. In addition, the dependence of *ℒum*_max,1_ on $c_{\mathrm{Cd}}^{*}$ in 0.1% LB is impacted by *x* to a smaller extent than in 0.1% tryptone. Figure 7 provides an overview of these comparative observations by reporting the slopes of *ℒum*_max,1_ (Figure 7A), *ℒum*_max,2_ (Figure 7B) and *ℒum_max_*_,3_ (Figure 7C) versus $c_{\mathrm{Cd}}^{*}$ for the various *x*-conditions tested in 0.1% tryptone, 0.1% LB and 0.15% LB. The main conclusions drawn from Figure 7 can be summarized as follows. First, the dependence of the slopes on *x* as estimated for the P1, P2 and P3 emission modes qualitatively follows the variation of the corresponding peak maxima with changing *x* (Figure 5, Figures S4 and S5). Quantitatively, the slopes pertaining to emission P1 at a fixed value of *x* conform to the sequence of 0.1% LB~0.1% tryptone < 0.15% LB, with the notation ‘Y < Z’ meaning ‘the slope estimated in medium Y is lower than that in medium Z under given *x* condition’; in the P2 emission mode, the slopes satisfy the sequence of 0.15% LB ≤ 0.1% LB ≤ 0.1% tryptone, and this sequence basically applies in the P3 emission. The respective magnitudes of the slopes relevant in P1 and P2 emissions in 0.1% and 0.15% LB media agree with the conclusions drawn from our previous work [32] on the stringence-controlled bimodal cell response and variations thereof with changing LB concentration. Finally, Figure 7B clearly highlights that the full suppression of peak P2 with decreasing glucose concentration occurs at a lower value of *x* in 0.1% tryptone compared to that observed in 0.1–0.15% LB situations.

Based on Equation (8), the sequence identified above for the slopes $\partial ℒum_{\max,1}/\partial c_{\mathrm{Cd}}^{*}$ in emission mode 1 (Figure 7A) where the biouptake of amino acids determines light production, can be understood as follows. Whereas Cd speciation is expected to be more significant in 0.15% LB than in 0.1% LB, the rate of increase in $ℒum_{\max,1}$ with $c_{\mathrm{Cd}}^{*}$ is larger in the former medium. Accordingly, we suggest from Equation (8) that it is the increase in the carrying capacity $c_{p,1}^{\max}$ from 0.1% to 0.15% LB that dominates the corresponding change in $\partial ℒum_{\max,1}/\partial c_{\mathrm{Cd}}^{*}$ rather than the expected increase in ${\bar{K}}_{s}$. The latter should, if solely considered, have led to a decrease in the slope $\partial ℒum_{\max,1}/\partial c_{\mathrm{Cd}}^{*}$ from 0.1% to 0.15% LB (see Equation (8)). The higher is the concentration in amino acids and the lesser the rate of light production becomes limited by this resource (i.e., the carrying capacity is higher), thus leading to an increase in $\partial ℒum_{\max,1}/\partial c_{\mathrm{Cd}}^{*}$ even though metal ions are expected to be lesser bioavailable in 0.15% LB. The relative magnitudes of $\partial ℒum_{\max,1}/\partial c_{\mathrm{Cd}}^{*}$ in 0.1% LB and 0.1% tryptone are quite comparable, especially at high values of *x*. Here, the loss in bioluminescence expected from the lower bioavailability of Cd ions in 0.1% LB due to a larger complexation (higher ${\bar{K}}_{s}$ in Equation (8)) than in 0.1% tryptone is counterbalanced by the higher quality of the provided food in the LB medium compared to that in tryptone (i.e., higher $c_{p,1}^{\max}$) due to the presence of a yeast extract that contains various amino acids, vitamins and minerals [46]. In turn, in qualitative agreement with Equation (8), both food quality and metal bioavailability features have to be advanced to understand the rather similar performance of the biosensors in 0.1% LB and 0.1% tryptone. Considering the stringence-controlled emission of light in mode 2, the sequence of slopes $\partial ℒum_{\max,2}/\partial c_{\mathrm{Cd}}^{*}$ is expected to be the invert of that discussed in mode 1. In line with [32], this expectation is verified (Figure 7B), at least for sufficiently large values of *x* (>0.2–0.3) for which there is no limitation in glucose for cells to produce light. For lower values of *x* where glucose-limitation clearly sets in with resulting decrease in $\partial ℒum_{\max,2}/\partial c_{\mathrm{Cd}}^{*}$ along with decreasing *x*, differences between slopes measured in 0.1% tryptone, 0.1% LB and 0.15% LB become less pronounced as cells gradually shift the required sugar source for amino acid metabolism from glucose (P2 peak) to xylose (P3 peak). This in turn explains the opposite evolutions of $\partial ℒum_{\max,2}/\partial c_{\mathrm{Cd}}^{*}$ and $\partial ℒum_{\max,3}/\partial c_{\mathrm{Cd}}^{*}$ with decreasing *x* (Figure 7B,C), while the slope sequence versus the 0.1% tryptone, 0.1% LB, and 0.15% LB conditions in emission mode 2 is roughly the same as that in mode 3.

### 4.3. Origin of the Observed Modulations of the Bioluminescence Signal with Time an Metal Concentration: Interplay between Stringent Response and Catabolite Repression

**Figure 8:**
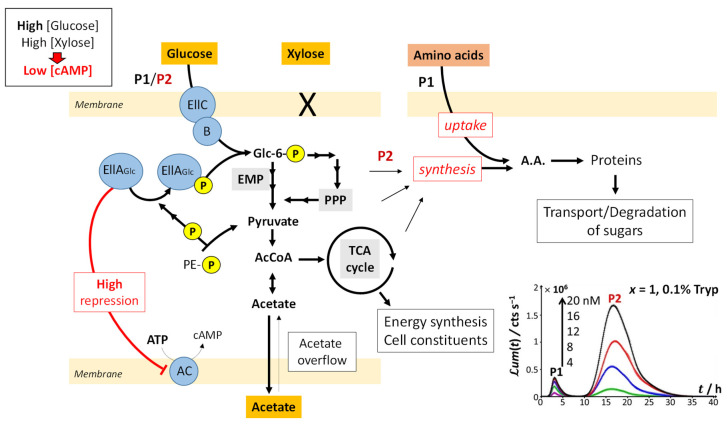
Schematic representation of the mechanisms underlying sugar degradation and amino acids uptake/synthesis in *E. coli* at sufficiently large glucose concentrations (high *x*), which leads to peaks P1 and P2. Under such *x*-conditions, glucose is transported into the cell by the joint action of the EIIC-B and EIIA-P proteins, the main components of the sugar phosphotransferase system together with Hpr and EI (not shown in the figure for simplicity). The EIIA-P proteins transfer a phosphate group (acquired from phosphoenolpyruvate) to glucose, which enters the TCA cycle as acetyl-CoA after degradation in the Embden–Meyerhof–Parnas (EMP) or pentose phosphate pathway (PPP). This leads to the production of the energy required to sustain light production in the emission modes 1 and 2 (P1 and P2 signals, respectively). Acetate from glucose breakdown is then expelled from the cell. As a result, unphosphorylated EIIA becomes more abundant, inhibits adenylate cyclase, and lowers cAMP levels, which does not allow for both the formation of the cAMP/Crp complex and the transcriptional activation of acetate and xylose operons. Light emission corresponding to the P1 peak is thus partly determined by the concentration of bioavailable amino acids. When there is a lack of amino acids, cells reallocate resources for the synthesis of amino acids, leading to the P2 peak. Abbreviations: PE-P, phosphoenolpyruvate; Glc-6P, Glucose 6-phosphate; PPP, the pentose phosphate pathway; AcCoA, acetyl CoA; TCA cycle, tricarboxylic acid cycle; AC, adenylate cyclase; ATP, adenosine triphosphate; cAMP, cyclic adenosine monophosphate; A.A., amino acids.

Figure 1, Figure 2, Figure 3 and Figure 4 discussed in Section 4.1 evidence marked modulations of the time-dependent bioluminescence response with varying medium composition under a given Cd concentration condition. These changes of the biosensor signal are intimately connected to the metabolic pathway at stake over time, depending on the bioavailability and nature of the resources in solution. In the presence of amino acids (either tryptone or LB) and a significant source of carbon brought by glucose (Figure 1A, Figures S2A and S3A), peak P1 in the bimodal P1–P2 signal follows the uptake of amino acids. Their ensuing depletion from solution triggers a stringent cell response that leads to peak P2, as detailed elsewhere [32]. In detail, Figure 8 illustrates the processes that drive glucose transport and metabolism in E. coli together with the amino acid biosynthetic pathway. The uptake of glucose is mediated by joint action of EIIC-B proteinaceous complex (which ensures glucose transfer across the membrane) and phosphorylated EIIA proteins (that acquire their P group from phosphoenolpyruvate, denoted as PE-P in Figure 8). EIIA-P proteins transfer a phosphate group to glucose that—after the Embden–Meyerhof–Parnas (EMP) pathway (glycolysis) or the pentose phosphate pathway (PPP)—enters the Krebs cycle (or TCA cycle), which produces the energy necessary for cells to emit light. Thus, the unphosphorylated EIIA proteins (having transferred their phosphate group) repress the adenylate cyclase (AC) and therefore inhibit the production of the cyclic adenosine monophosphate (cAMP), a mediator of the catabolite repression [47]. In the E. coli glucose metabolic pathway, several precursors for amino acid biosynthesis are primarily generated and a large part of carbon incorporated as glucose is catabolized and excreted as acetate into the medium according to a phenomenon known as acetate overflow [48].

**Figure 9:**
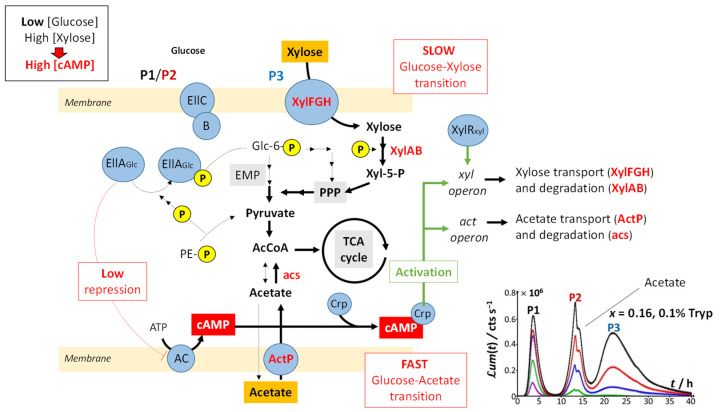
Schematic representation of the mechanisms underlying sugar degradation at sufficiently low glucose concentrations (low *x*), which leads to the appearances of the P2 shoulder/P2 doublet and P3 peak. Under such *x*-conditions, glucose is transported into the cell and degraded to produce the energy required for light emission in modes 1 and 2 (P1 and P2, respectively), as detailed in Figure 8. In parallel, acetate originating from glucose breakdown is expelled from the cell. A low glucose concentration leads to a lower amount of non-phosphorylated EIIA proteins and thus to a lower inhibition of adenylate cyclase. The concentration level of cAMP thus increases, which allows for the formation of the cAMP/Crp complex and the activation of the simultaneous transcriptions of the acetate and xylose operons (albeit at different rates) necessary for their intracellular transport and degradation. Abbreviations: PE-P, phosphoenolpyruvate Glc-6P; Glucose 6-phosphate; EMP, Embden–Meyerhof–Parnas pathway; PPP, the pentose phosphate pathway; AcCoA, acetyl CoA; TCA cycle, tricarboxylic acid cycle; AC, adenylate cyclase; ATP, adenosine triphosphate; cAMP, cyclic adenosine monophosphate; Crp, cAMP receptor protein; ActP, cation/acetate symporter. The inset displays the time-dependent luminescence response corresponding to the molecular mechanisms highlighted in the scheme, with the P2 shoulder originating from the acetate that is metabolized by cells.

Upon gradually replacing glucose by xylose (decreasing values of x), three main signal features are observed: truncation of peak P2, followed over time by the appearance of a shoulder on peak P2 and the detection of a third bioluminescence production mode (peak P3). Whereas P2 truncation originates from glucose depletion in solution (the more so as glucose concentration decreases; see Figure 2B,C), the shoulder on peak P2 and the appearance of peak P3 are the results of catabolite repression. In detail, Figure 9 illustrates the mechanisms underlying the catabolite glucose/acetate and glucose/xylose repressions at the origin of the shoulder on peak P2 and the appearance of peak P3, respectively. For cases where glucose deficiency is sensed by the cells and xylose is present in the medium, EIIA proteins then become mainly present in the phosphorylated form and AC repression is turned off. Thus, cAMP and Crp levels increase, which activates *act* and *xyl* operons via the formation of the cAMP/Crp complex. Consequently, both acetate (previously expelled by the cell) and xylose enter the cell via ActP and XylFGH proteins, respectively. Acetate reaches TCA cycle after degradation by an acetate synthetase (acs), whereas xylose successively enters the PPP, EMP pathway and TCA cycle after degradation via the enzymes XylA (xylose isomerase) and XylB (xylose kinase). This translates into the transition from truncated peak P2 (Figure 2C) to truncated P2 peak featuring a shoulder and into the appearance of peak P3 (Figure 2D–H) as a result of expelled acetate and xylose catabolism, respectively. It is stressed that the degradation of acetate occurs prior to that of xylose even though the expression of the *act* operon is concomitant to that of *xyl* operon (see the sequential appearance of the P2 shoulder and P3). This phenomenon is related to the short adaptation phase of glucose-acetate transition (i.e., 10–40 min) compared to that of the glucose-xylose (i.e., 1.8–5.8 h) in *E. coli* [49,50]. When the glucose concentration becomes too low, the P2 peak disappears due to resulting insufficient amount of energy to sustain light production in emission mode 2. The truncation and shoulder observed previously on the P2 signal then appear on the P1 signal, which leads to a decrease in its magnitude (Figure 2I–L). Under these conditions, the stringent response and catabolite repression occur simultaneously.

The increase in *ℒum*_max,1_ with decreasing glucose concentration from *x* = 1 to 0.2 and the presence of local maxima for *ℒum*_max,1_ at *x* = 0.16 and 0.06 (Figure 5A) suggest that the hierarchical utilization of sugars (as possibly inferred from the sequential appearance of P2 shoulder and peak P3) does not proceed according to an ‘on/off’ process. Instead, cells likely use the different carbon sources at their disposal depending on demand and energy trade-offs (e.g., use of glucose/acetate or glucose/xylose for 0.06 < *x* < 1 and use of glucose/xylose/acetate under conditions corresponding to local maxima in *ℒum*_max,1_), as shown in Figure 9. In agreement with our observations, several studies have evidenced that bacterial response to changes in energy supply should be viewed as a continuous process rather than a threshold phenomenon, especially in carbon-limited cultures [51,52]. When glucose concentration is sufficiently low, the cAMP level increases and the cAMP–Crp complex can then simultaneously induce the transcriptions of both acetate and xylose operons and the synthesis of the enzymes necessary for their respective catabolism. Most importantly, the rate of transcriptions of these two operons may differ (Figure 9), which agrees with a continuous rather than an ‘on/of’ use of distinct carbon sources.

Another possibility, supported by the recent observations by Barthe et al. [50], is the existence of subpopulations of cells during their adaptation to a transition from glucose to xylose carbon sources. The related phenotypic heterogeneity in the use of substrates during the diauxic batch culture of a monoclonal population is related to the intracellular availability of XylR that controls the length of catabolite repression phase and therefore mediates the emergence of subpopulations able to handle xylose [50]. This mechanism leads to a transition phase where both glucose and xylose may be jointly exploited by distinct cell populations.

### 4.4. On the Connection between Bioluminescence Response of Metal-Sensing Cells, Cell Photoactivity and Nutrient Conditions

**Figure 10:**
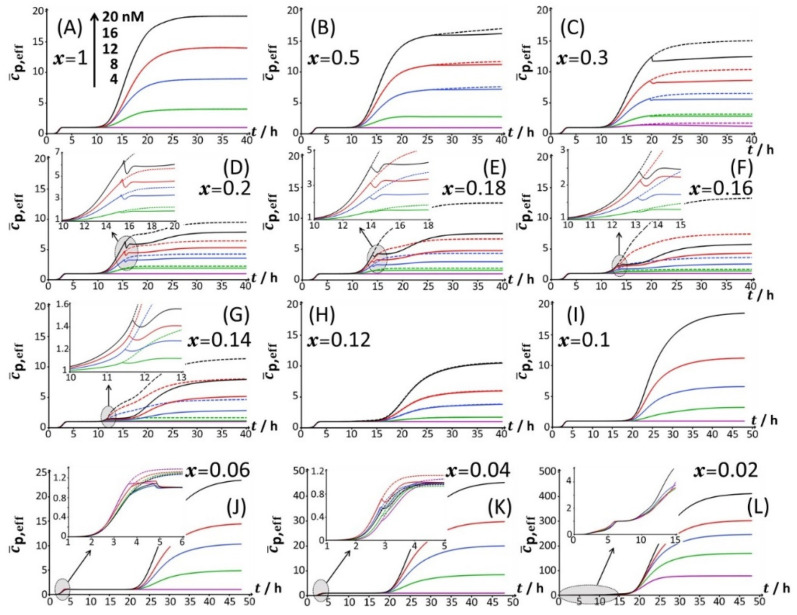
Dependence of the cell photoactivity ${\bar{c}}_{p,\mathrm{eff}}$ (Equation (10)) on time *t* and total bulk Cd concentration (specified) in 0.1% tryptone for different glucose-to-xylose concentration ratios corresponding to 0.02 ≤ *x* ≤ 1 (indicated). The scale in Cd concentration and the associated color nomenclature specified in (**A**) apply to panels (**B**–**L**). Solid lines: cell photoactivity retrieved from the theoretical reconstruction of the measured time-dependent bioluminescence profiles displayed in Figure 2 following the methodology delineated in Section 3 (Equations (9) and (10)). Dotted lines correspond to predictions in the absence of truncation in peak P2 (panels (**B**–**G**)) and in peak P1 (panels (**J**,**K**)). The theoretical time-dependent bioluminescence patterns corresponding to the ${\bar{c}}_{p,\mathrm{eff}}$ data given in this Figure 10 are shown in Figure 2 (solid lines therein) and in Figure 3 and Figure 4 where zooms of the P2 and P1 emission modes, respectively, are provided.

Using the theory detailed in Section 3.1 and following the methodology for bioluminescence signal analysis described in Section 3.2, the time-dependent bioluminescence response of metal-sensing cells measured in 0.1% tryptone (Figure 2, Figure 3 and Figure 4) could be successfully reconstructed for all tested Cd concentrations and glucose-to-xylose concentration ratios. Supplementary Table S1 specifies values of the root mean square error (RMSE) underlying the quality of the theoretical reconstructions of the measured bioluminescence signals for 0 < *t* < 48 h at all values of *x* and total Cd concentration adopted in this work. The fitting of the bioluminescence profiles to theory is reported in Figure 2, Figure 3 and Figure 4 (solid lines therein), and the corresponding dependence of the dimensionless cell photoactivity on time, ${\bar{c}}_{p,\mathrm{eff}}\left(t\right)$ (Equation (10)) as retrieved from fitting of the cell response to Equation (9), is given in Figure 10 for each examined condition. For the sake of completeness, we mention that successful confrontations between theoretical formalism and bioluminescence signals monitored in 0.15% and 0.1% LB media were also achieved (solid lines in Figures S2 and S3), and related time-dependent cell photoactivity patterns are shown in Figures S7 and S8 for all tested values of *x* and $c_{\mathrm{Cd}}^{*}$. At a fixed Cd concentration and *x*, the sequential appearance of *m* (non-truncated and non-doubled) bioluminescence peaks (with *m* = 1, 2 or 3 corresponding to peaks P1, P2 and/or P3 depending on *x*) was reflected by a ${\bar{c}}_{p,\mathrm{eff}}\left(t\right)$ that increases with time according to *m* successive sigmoid-like functions. The latter functions feature non-zero plateau values marking the transition between two successive peaks, and the plateau ${\bar{c}}_{p,\mathrm{eff}}\left(t\to \infty \right)$ corresponds to the bioluminescence response at t→∞ where ℒum(t→∞)→0. These properties are the direct consequence of the convolution product that defines ℒum(t) (Equation (1)), and they further agree with the ${\bar{c}}_{p,\mathrm{eff}}\left(t\right)$ features revealed for bimodal stringent-controlled signals [32]. This convolution product finds its physical origin in the finite timelapse (denoted as $\tau_{q}$ in Section 3), during which an excited luciferase–hydroxyflavin complex can emit photons before its rate of photon production goes to 0 [14]. The resulting bioluminescence at time *t* is then proportional to the time-dependent concentration of photon emitters between 0 and *t* (emitters being operational during a timelapse $\tau_{q}$), and this concentration varies with time according to ${\bar{c}}_{p,\mathrm{eff}}\left(t\right)$. Of interest here is the way the truncation of peak P2 (see Section 4.2) resulting from the glucose-mediated repression of acetate (Section 4.3) is reflected in the ${\bar{c}}_{p,\mathrm{eff}}\left(t\right)$ profile. Figure 10C clearly shows that for a bimodal P1-truncated P2 signal (Figure 2C), the truncation of peak P2 leads to a discontinuous decrease in ${\bar{c}}_{p,\mathrm{eff}}\left(t\right)$ with time followed by a plateau value reached when bioluminescence approaches a value of 0. When there is repression of acetate by glucose and subsequent use of acetate by the cells (Figure 2D), the corresponding truncation of peak P2 and the appearance of a shoulder in that peak give rise to a local minimum in ${\bar{c}}_{p,\mathrm{eff}}\left(t\right)$, as pictured in the inset of Figure 10D. The depth of that minimum further increases with increasing $c_{\mathrm{Cd}}^{*}$. Similar features apply for ${\bar{c}}_{p,\mathrm{eff}}\left(t\right)$ corresponding to a truncated P1 signal (as observed at sufficiently low *x*, see Figure 2J,K and Figure 4B,C) due to early catabolite repression (Figure 10J,K). The effects of truncation of P2 and P1 peaks on ${\bar{c}}_{p,\mathrm{eff}}\left(t\right)$ can be further appreciated in Figure 10 by comparing the theoretical results derived from proper reconstruction of the bioluminescence signal (solid lines) with those generated for the scenario where peak truncation is absent. Concretely, the latter results are obtained by replacing the time component of ${\bar{c}}_{p,\mathrm{eff}}\left(t\right)$ adopted to recover the observed truncation by a value of 0.

**Figure 11:**
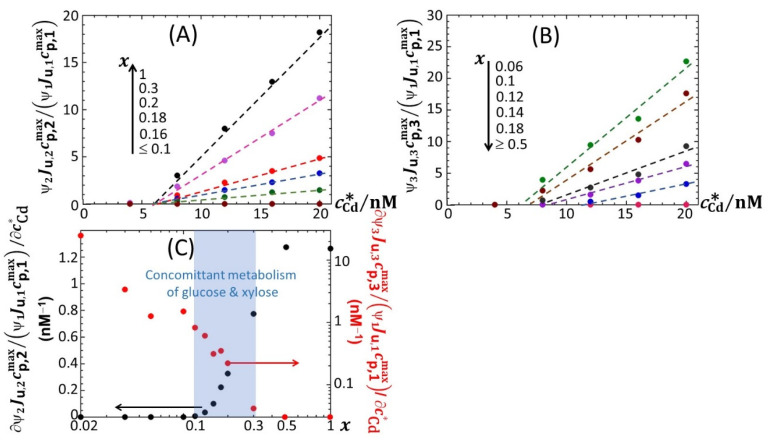
(**A**,**B**) Dependence of the ratios $R_{i=2,3}=\frac{\Psi_{i=2,3}J_{u,i=2,3}c_{p,i=2,3}^{\max}}{\Psi_{1}J_{u,1}c_{p,1}^{\max}}$ on $c_{\mathrm{Cd}}^{*}$ for selected values of *x* in 0.1% tryptone. (**C**) Slopes of the linear variations of $R_{i}$ with $c_{\mathrm{Cd}}^{*}$ reported as a function of *x* in 0.1% tryptone. The blue-shaded zone identifies the range of *x* values where cells concomitantly metabolize glucose and xylose. Data in this figure originate from those given in Figure 2, Figures S2 and S3 (corrected for the corresponding reference at 0 nM Cd concentration).

Now that the overall dependence of ${\bar{c}}_{p,\mathrm{eff}}\left(t\right)$ on glucose concentration *x* has been discussed in connection with time-dependent bioluminescence profiles and the modality/truncation thereof, we comment on the dependence of ${\bar{c}}_{p,\mathrm{eff}}\left(t\right)$ on $c_{\mathrm{Cd}}^{*}$ at selected values of *t* and *x*. For *x* ≥ 0.1, the first sigmoidal component of ${\bar{c}}_{p,\mathrm{eff}}$ corresponding to emission mode 1 (denoted as ${\bar{c}}_{p,1}\left(t\right)$ in Equation (10)) is found to not depend on $c_{\mathrm{Cd}}^{*}$. This finding directly follows Equations (9) and (10) for scenarios where cell photoactivity in emission mode 1 is not significantly affected by Cd ions in solution via, e.g., Cd-mediated hormetic or toxic effects. It further supports the applicability of the linear Henry regime for metal biouptake flux and that of the linear Hill regime for the production of reporter proteins, both being considered in the derivation of Equation (1) [14]. The applicability of these regimes ensure indeed that bioluminescence at any time *t* (which includes *ℒum*_max,i = 1,2,3_) linearly grows with $c_{\mathrm{Cd}}^{*}$ unless ${\bar{c}}_{p,\mathrm{eff}}\left(t\right)$ is affected by $c_{\mathrm{Cd}}^{*}$ following hormesis or toxicity effects. It is only for cases where truncation of peak P1 is observed that ${\bar{c}}_{p,\mathrm{eff}}\left(t\right)$ is significantly impacted by $c_{\mathrm{Cd}}^{*}$ (Figure 10J,K). For such poorly nutritive conditions marked by an early catabolite repression, the energy demands by the cells are not properly satisfied to sustain the reporter gene expression at a maximal rate, recalling that the latter increases with $c_{\mathrm{Cd}}^{*}$. Again, this result illustrates the subtle interplay between metal bioavailability in solution and the required bioavailability of nutrients for the cells to efficiently translate the supplied chemical information (i.e., the bioaccumulated amount of metal ions) into light. This point is further strengthened by examining the dependence of the dimensionless ratios $\frac{\Psi_{i=2,3}J_{u,i=2,3}c_{p,i=2,3}^{\max}}{\Psi_{1}J_{u,1}c_{p,1}^{\max}}$ (Equation (10)) on $c_{\mathrm{Cd}}^{*}$ and *x* (Figure 11A,B). To simplify notations, we hereafter denote this ratio as $R_{i=2,3}$. Qualitatively, the changes in $R_{i=2,3}$ with varying *x* at fixed $c_{\mathrm{Cd}}^{*}$ are found to mirror those observed for *ℒum*_max,i = 1,2,3_ in Figure 6. Namely, the decrease (increase) in $R_{2}$ ($R_{3}$, respectively) with decreasing *x* accompanies the gradual vanishing and appearance of peaks P2 and P3, respectively, considering that the corresponding variations of bioluminescence in emission mode 1 (when it is significantly operative, i.e., for 0.06 < *x* < 1) at fixed $c_{\mathrm{Cd}}^{*}$ are less pronounced than those in modes 2 and 3 (Figure 6). We further found that $R_{i=2,3}$ linearly increase at fixed value of *x* with increasing $c_{\mathrm{Cd}}^{*}$ above the minimal Cd-concentration values required for light induction and identified in Figure 6B,C. This result contrasts with the increase in log($R_{2}$) with $c_{\mathrm{Cd}}^{*}$ measured in LB media for another *lux*-CDABE biosensor constructed from the JW3596 *E. coli* strain [32]. Assuming that the ratios $\left(\Psi_{i=2,3}J_{u,i=2,3}\right)/\left(\Psi_{1}J_{u,1}\right)$ do not significantly depend on $c_{\mathrm{Cd}}^{*}$ (see definitions of $\Psi_{i=1,2,3}$ and $J_{u,i=1,2,3}$ given in Section 3), the observed linearity of $R_{i=2,3}$ with $c_{\mathrm{Cd}}^{*}$ then necessarily stems from a linear increase in $c_{p,i=2,3}^{\max}/c_{p,1}^{\max}$ with increasing $c_{\mathrm{Cd}}^{*}$ at fixed *x*. Stated differently, the medium can sustain a maximum size of photoactive cell population (the medium carrying capacity) in emission modes 2–3 that more strongly increases with $c_{\mathrm{Cd}}^{*}$ than the carrying capacity in mode 1 does. For the sake of completeness, the $\partial R_{i}/\partial c_{\mathrm{Cd}}^{*}$ slopes are reported in Figure 11C versus *x*. As discussed in Section 4.3, these data support the co-utilization of glucose and xylose by the biosensors in the range of 0.1 < *x* < 0.3. Analogous conclusions can be drawn for 0.15% and 0.1% LB media (Figures S9 and S10, respectively).

**Figure 12:**
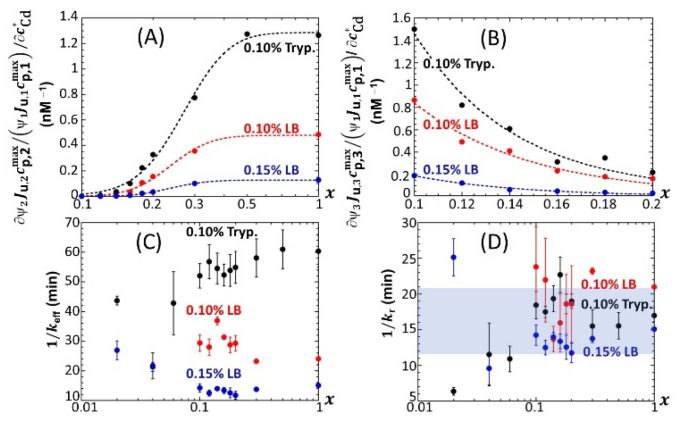
(**A**,**B**) Slopes of the linear variations of $R_{i=2,3}=\frac{\Psi_{i=2,3}J_{u,i=2,3}c_{p,i=2,3}^{\max}}{\Psi_{1}J_{u,1}c_{p,1}^{\max}}$ with $c_{\mathrm{Cd}}^{*}$ reported as a function of *x* in 0.1% tryptone, 0.1% LB and 0.15% LB media (indicated). Values of the timescales (**C**) $1/k_{\mathrm{eff}}$ and (**D**) $1/k_{r}$ as retrieved from recovery of measured time-dependent bioluminescence profiles (corrected for the corresponding reference measured at 0 nM concentration) with theory over the range of *x* conditions tested in 0.1% tryptone, 0.1% LB and 0.15% LB media (indicated). See text and Section 3 for details.

For the sake of comparison, Figure 12A,B shows the slopes $\partial R_{i=2,3}/\partial c_{\mathrm{Cd}}^{*}$ as a function of *x* for 0.1 ≤ *x* ≤ 1 in 0.1% tryptone, 0.1% LB and 0.15% media. The new feature revealed here is a decrease in the biosensor performance (at any *x*) from mode 1 to mode 2 (Figure 12A) and from mode 1 to mode 3 (Figure 12B) upon replacing the 0.1% tryptone medium with a 0.1% LB or 0.15% LB medium. Among the three tested media, the 0.15% LB solution is the one with the highest nutritional quality for cells that operate in mode 1 [46]. This is materialized by values of $\partial ℒum_{\max,1}/\partial c_{\mathrm{Cd}}^{*}$ that are highest in 0.15% LB under all $c_{\mathrm{Cd}}^{*}$ and *x* conditions tested (Figure 7A). As a result, unlike in 0.1% LB or 0.1% tryptone, a gain in biosensor performance when passing from mode 1 to mode 2 or to mode 3 becomes more difficult to achieve in the 0.15% LB medium that already features the best biosensor performance in mode 1. In turn, the increase in the carrying capacity ratios $c_{p,i=2,3}^{\max}/c_{p,1}^{\max}$ with $c_{\mathrm{Cd}}^{*}$ (which determines $\partial R_{i=2,3}/\partial c_{\mathrm{Cd}}^{*}$ provided that $\left(\Psi_{i=2,3}J_{u,i=2,3}\right)/\left(\Psi_{1}J_{u,1}\right)$ does not depend on $c_{\mathrm{Cd}}^{*}$; see argument above) is more important in 0.1% tryptone than it is in 0.1% and 0.15% LB. Interestingly, the timescale $1/k_{\mathrm{eff}}$ (Equation (7)) evaluated from the modeling of the bioluminescence signals (Section 3.2) is found to significantly decrease at any *x* when shifting from 0.1% tryptone to 0.15% LB (Figure 12C), i.e., when significantly increasing the rate of light production per unit metal concentration in mode 1 (Figure 7A) (it is in this mode that $1/k_{\mathrm{eff}}$ is estimated; see Section 3.2). This observation qualitatively conforms with the dependence $1/k_{\mathrm{eff}}\propto 1/k_{e}$ evidenced by Equation (7). Indeed, a higher rate of light production is associated with a higher rate of transcription of zntA that codes a P-type ATPase pump involved in Cd efflux. This in turn facilitates Cd excretion, which comes to effectively increase $k_{e}$ and therefore decrease $1/k_{\mathrm{eff}}$. This feature, however, cannot explain on its own the significant decrease in $1/k_{\mathrm{eff}}$ when moving from 0.1% tryptone to 0.1% LB (Figure 12C) because the $\partial ℒum_{\max,1}/\partial c_{\mathrm{Cd}}^{*}$ achieved in these media is of the same order of magnitude (Figure 7A). This latter decrease in $1/k_{\mathrm{eff}}$ from the 0.1% tryptone to 0.1% LB media can be understood from Equation (7) and the dependence of $1/k_{\mathrm{eff}}$ on Cd speciation features in solution via the quantity *p* that ranges from unity for inert metal complexes to $\left(1+\varepsilon {\bar{K}}_{s}\right)$ for fully labile complexes. Indeed, Cd binding to charged molecules or particles is known to be essentially driven by (attractive) electrostatics, which favors the lability of complexes with faster interconversion between free and complexed metal forms [53]. Given the respective compositions of LB and tryptone media, it is further expected that the stability constant ${\bar{K}}_{s}$ (which involves the concentration of ligand) would be higher in 0.1% LB than in 0.1% tryptone. In turn, the parameter *p* would likely be larger in the former medium, which would lead to a lower $1/k_{\mathrm{eff}}$ (see Equation (7)), in line with data in Figure 12C. In practice, both impacts of medium composition on $k_{e}$ and *p* discussed above probably jointly determine $1/k_{\mathrm{eff}}$. This discussion, even qualitative, may suggest plausible leading-order mechanisms consistent with the $1/k_{\mathrm{eff}}$ sequence displayed in Figure 12C at fixed *x*. Within the error bars, $1/k_{\mathrm{eff}}$ does not significantly vary with decreasing *x* from unity to 0.1, and values corresponding to *x* < 0.1 should be considered with caution due to the truncation or even doubling of peak 1 (Figure 4), which increases the required parametrization level of ${\bar{c}}_{p,1}\left(t\right)$ at *x* < 0.1. The error bars in Figure 12C represent the dispersion in $1/k_{\mathrm{eff}}$ values estimated over the 0–20 nM range in Cd concentration: no obvious dependence of $1/k_{\mathrm{eff}}$ on $c_{\mathrm{Cd}}^{*}$ can be identified, and a similar conclusion holds for $1/k_{r}$, in line with results reported elsewhere [32]. For 0.1 < *x* < 1, the magnitude of $1/k_{r}$ (~10–20 min) found for the three tested media is consistent with that given in our previous work conducted on another *lux*-CDABE *E. coli* biosensor in a medium lacking xylose [32]. This magnitude is further in agreement with the half-life of luciferase proteins reported by Koga et al. from in vitro experiments [54].

## 5. Conclusions

In this work, we provide a full analysis of the bioluminescence signals produced over time by Cd-detecting *luxCDABE E. coli* sensors in media where glucose carbon source is gradually replaced by xylose in the presence of amino acids brought by tryptone or LB. Under conditions where the glucose resource is not limited, the bioluminescence signal exhibits a sequence of two successive bell-shaped peaks P1 and P2 resulting from amino acid absorption and stringent cell response, respectively. Upon setting the biosensors in media where the carbon source is significantly shifted from glucose to xylose, a clear truncation of the peak P2 is observed as a result of acetate repression by glucose. Under such conditions, xylose repression by glucose is also operational. A doubling of peak P2 is even observed when the shortage in glucose is so severe that the repression of acetate is stopped and acetate becomes actively metabolized by the cells in replacement of glucose. These features are accompanied by a progressive extinction of peak P2 with decreasing glucose concentration below a threshold value (i.e., with significantly increasing xylose concentration) and by the joint appearance (at longer timescales) of a third peak P3 due to the utilization of xylose after the cells have ended their xylose-mediated repression effective at larger glucose concentrations. The resulting P1–P3 bimodal signal with xylose as the carbon source then constitutes the pendant of the P1–P2 bimodal signal triggered by the stringent cell response in media containing glucose in excess over xylose. The truncation, doubling and extinction of peak P1 are observed with further increasing xylose concentration as glucose concentration then becomes too low to sustain amino acid absorption and subsequent light production. The respective biosensor performances estimated from the rates of increase in the detected peak maxima with increasing Cd concentration are further discussed as a function of glucose-to-xylose concentration ratio and expected differences in metal speciation in tryptone- and LB-containing media. The analysis highlights the requirement to invoke both nutrient bioavailability/quality and metal bioavailability for a qualitative capture of the respective dependence of the bioluminescence signals on Cd concentration within the emission modes corresponding to peaks P1, P2 and P3. The discussion is further supported by a successful confrontation between measured bioluminescence data and theoretical formalism, as well as the subsequent derivation of the time-dependent cell photoactivity profiles for all examined media compositions. The analysis again highlights the intrinsic dual components of the bioluminescence response, i.e., metal bioavailability properties in solution, and supply by the medium of the energy required for cells to sustain metal-triggered light production. Based on the reported findings, we may state that the detection of Cd^2+^ by the biosensor of interest in this work is best performed upon analysis of the response in the P1–P2 (bimodal signal), P1–P2–P3 (trimodal signal) or P1–P3 (bimodal signal) configurations depending on nutritional medium quality. In support of this recommendation, our results illustrate how the lower detection limit (or sensitivity) can be tuned when switching from P1 to P2/P3 peak signals and how the amplitude of the considered peak (and therefore the signal-to-noise ratio) can be changed from one mode of emission to the other. In turn, a concomitant exploitation of these properties and of the linear relationships established between peak amplitude and metal concentration offers a panel of options to efficiently track Cd^2+^ at an optimized quantitative level. This is clearly an advantage over current practice where metal detection is often addressed from the only exploitation of a single bioluminescence peak signal.

Though the focus of this study is on the effects of nutritional conditions on the multimodality of bioluminescence emission patterns, forthcoming contribution from our group will delineate a strategy to connect (on a quantitative level) metal bioavailability properties and the time–response of luminescent metal whole-cell biosensors without the recourse to calibration protocol and without the common but questionable assumption of Biotic Ligand Model validity. As shown in the current study, the method will include the necessary estimation of cell photoactivity dependence on time via a proper normalization and time deconvolution of the cell signal.

This study demonstrates the proof-of-concept for modifying on demand bioluminescence signals generated by whole-cell metal biosensors upon exploiting the dependence of cell photoactivity on nutrient medium conditions. The modifications apply to the number, position, shape and magnitude of the bioluminescence peaks sequentially measured over time, each peak being the characteristic signature of a specific cell metabolism. In turn, these results open perspectives in terms of the detection of the toxicity of contaminants by metal-responsive bioluminescent sentinels as a function of pristine cell metabolisms. Such perspectives should help to extend current practice mostly restricted to the measurement of the decay in bioluminescence signal amplitude arbitrarily selected at a fixed timepoint with increasing toxicant concentration.

## Data Availability

All raw bioluminescence data reported in this work are available upon request, as is the PTC Mathcad Prime code developed for the theoretical analysis of the time-dependent bioluminescence response.

## Supplementary Materials

The following supporting information can be downloaded at: https://www.mdpi.com/article/10.3390/bios12050327/s1. In the Supporting Information, we provide the equivalents of Figure 2, Figure 5, Figure 6, Figure 10 and Figure 11 for 0.10% and 0.15% LB media (Figures S2–S10); in Figure S1, we provide an illustrative bioluminescence dataset that is uncorrected for the reference signal measured in the absence of Cd; and in Table S1, we provide the root mean square error values associated with the fitting of the data in Figure 2 to Equations (9) and (10). All raw bioluminescence data reported in this work are available upon request, as is the PTC Mathcad Prime code developed for the theoretical analysis of the time-dependent bioluminescence response.
