# Supplementary material for: Exploiting Catabolite Repression and Stringent Response to Control Delay and Multimodality of Bioluminescence Signal by Metal Whole-Cell Biosensors: Interplay between Metal Bioavailability and Nutritional Medium Conditions
Source: Biosensors (Basel). 2022 May 11;12(5):327. doi: 10.3390/bios12050327 (PMC9139025; doi:10.3390/bios12050327)
Supplement: Supplementary file 1 [file biosensors-12-00327-s001.zip › biosensors-1718124-supplementary.pdf]

Supplementary Materials

Exploiting Catabolite Repression and Stringent Response to Control Delay and Multimodality of Bioluminescence Signal by Metal Whole-Cell Biosensors: Interplay between Metal Bioavailability and Nutritional Medium Conditions

Eva Delatour [1], Christophe Pagnout [1], Marie Zaffino [1] and Jérôme F.L. Duval [2,]*

[1] Université de Lorraine, CNRS, LIEC (Laboratoire Interdisciplinaire des Environnements Continentaux), UMR7360, Campus Bridoux, F-57070 Metz, France

[2] Université de Lorraine, CNRS, LIEC, UMR7360, F-54501 Vandoeuvre-lès-Nancy, France

*Correspondence: jerome.duval@univ-lorraine.fr

This document contains 10 Supplementary Figures and 1 Supplementary Table.

Supplementary figures (Figures S1–S10).

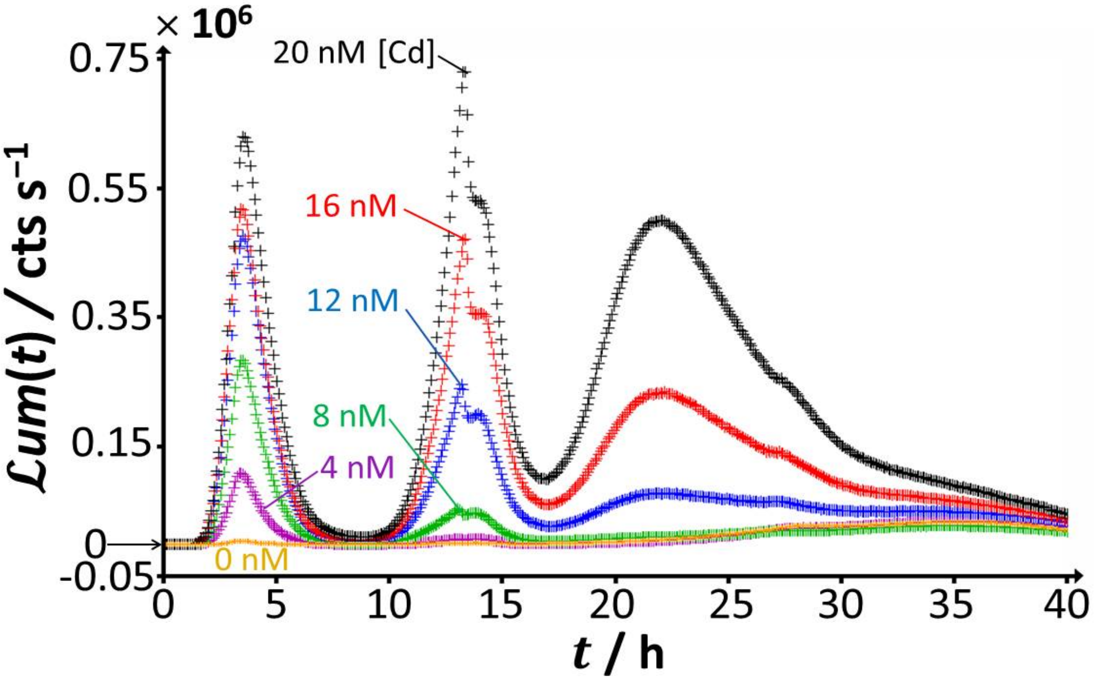

Figure S1. Time dependence of bioluminescence (*ℒum*(*t*) in counts s^{-1}) measured for the Cd-responsive *E. coli* biosensor as a function of total Cd concentration in the range 0-20 nM (indicated) in nGGM media supplemented with 0.1% tryptone and with concentrations of glucose and xylose correspondong to *x*=0.16. In this figure, biolumineseсence signals are **not** corrected for the cell response measured in 0 nM Cd.

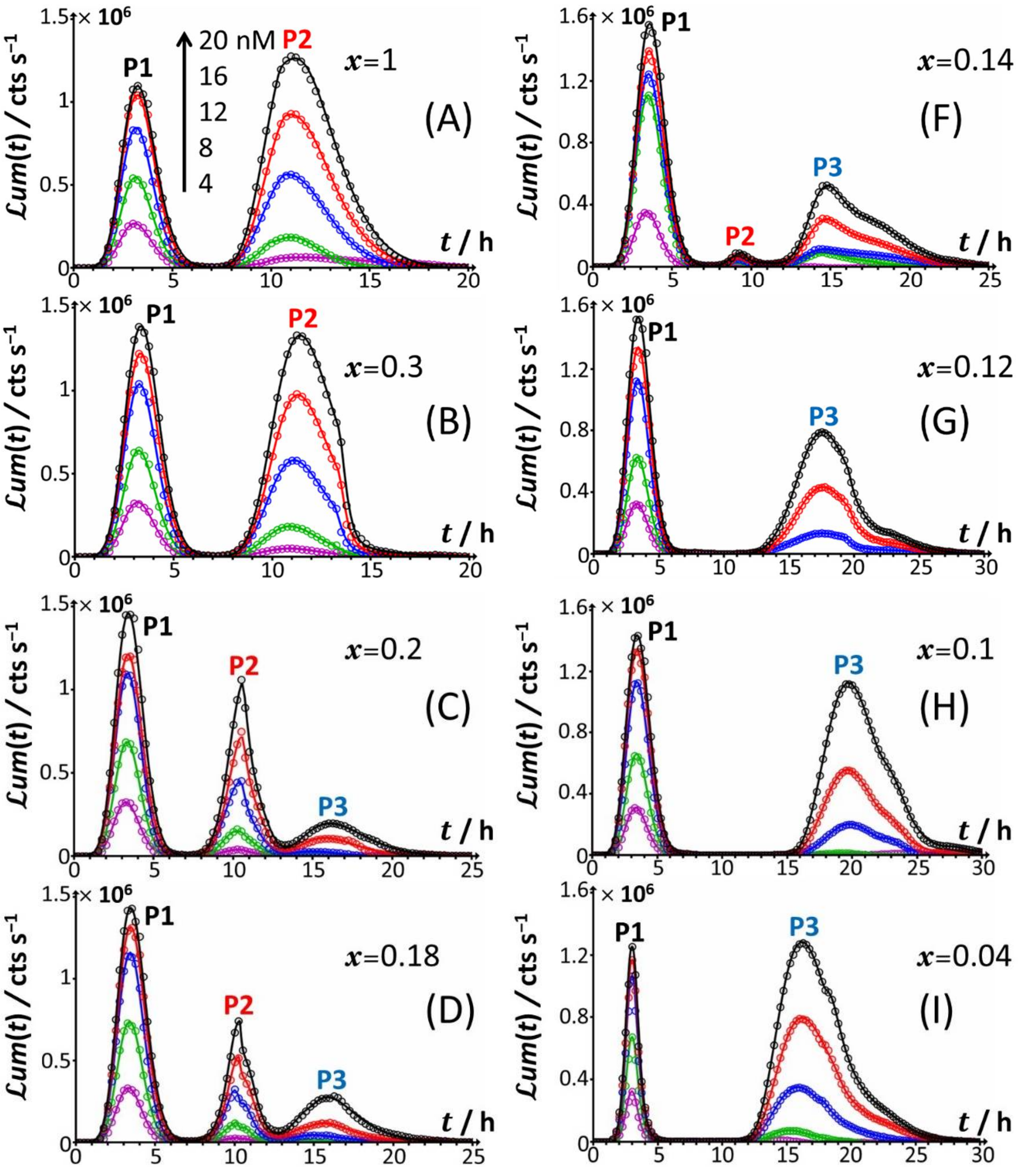
(A)
x=1
20 nM
16
12
8
4
P1
P2
(B)
x=0.3
(C)
x=0.2
P3
(D)
x=0.18
(F)
x=0.14
(G)
x=0.12
(H)
x=0.1
(I)
x=0.04
ℒum(t) / cts s⁻¹
t / h
× 10⁶

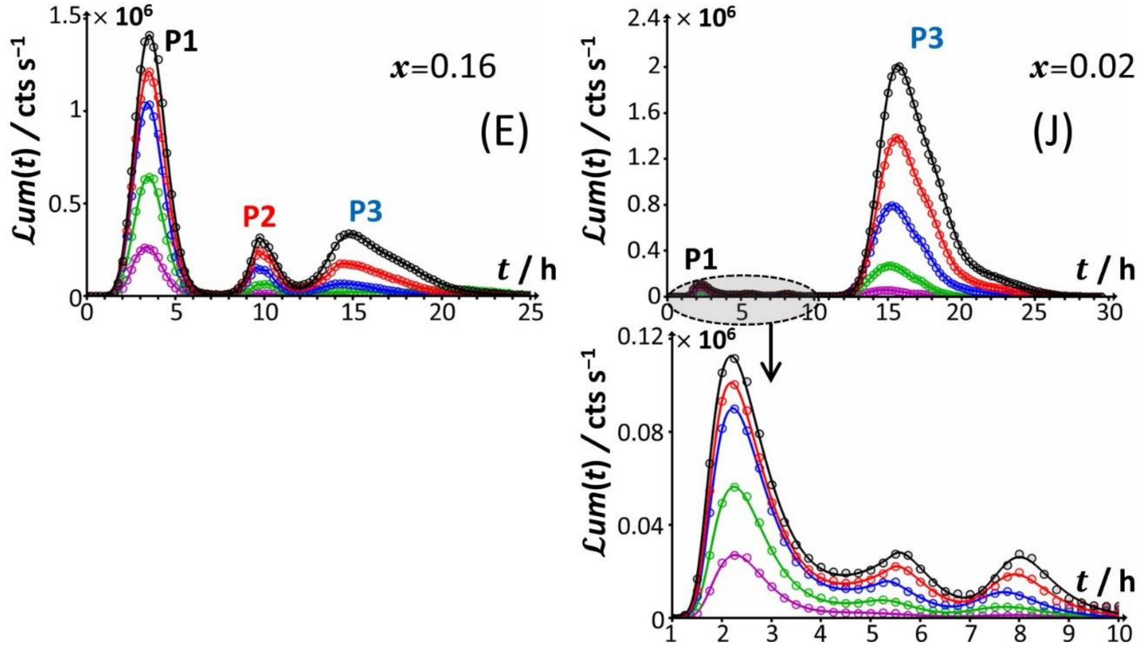

Figure S2. Time dependence of bioluminescence (*Lum*(*t*) in counts s^{-1}) measured for the Cd-responsive *E. coli* biosensor as a function of total Cd concentration in the range 0-20 nM (indicated) in nGGM media supplemented with 0.15% LB and with different concentrations of glucose and xylose, subsumed in the variable x with $0.02 \leq x \leq 1$. The scale in Cd concentration and associated color nomenclature specified in **(A)** apply to panels **(B)** to **(J)**. Symbols (o): experimental data. Lines: recontruction on the basis of the theory outlined in §3 of the main text. Signals are corrected by point-by-point substraction of the corresponding cell response measured in 0 nM Cd concentration.

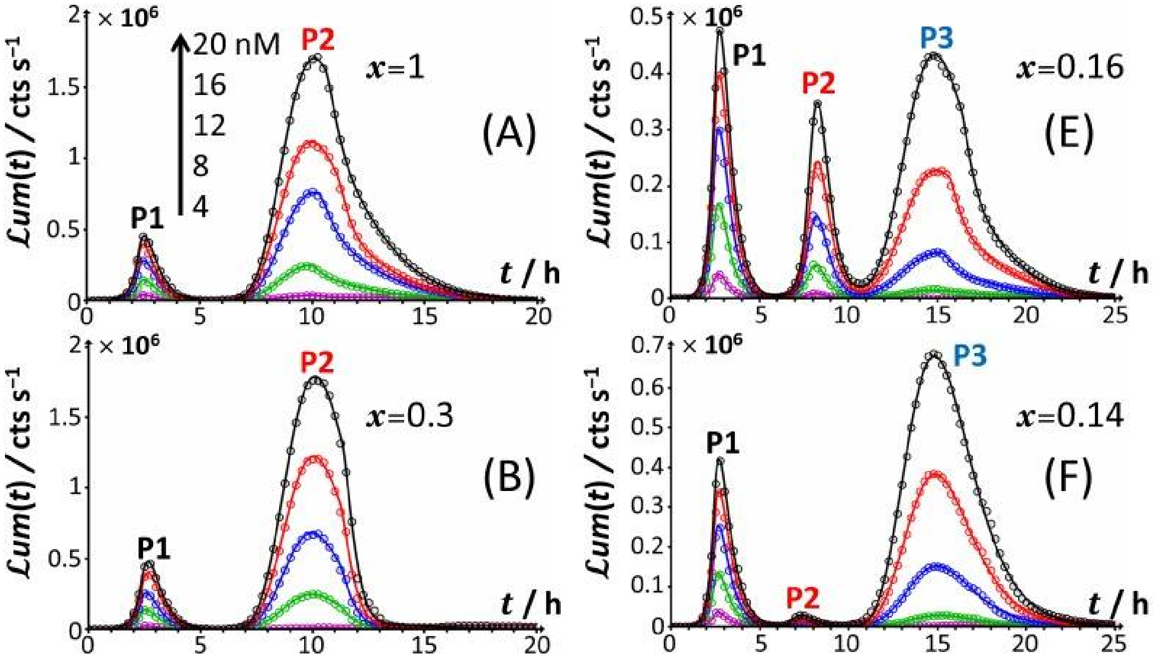

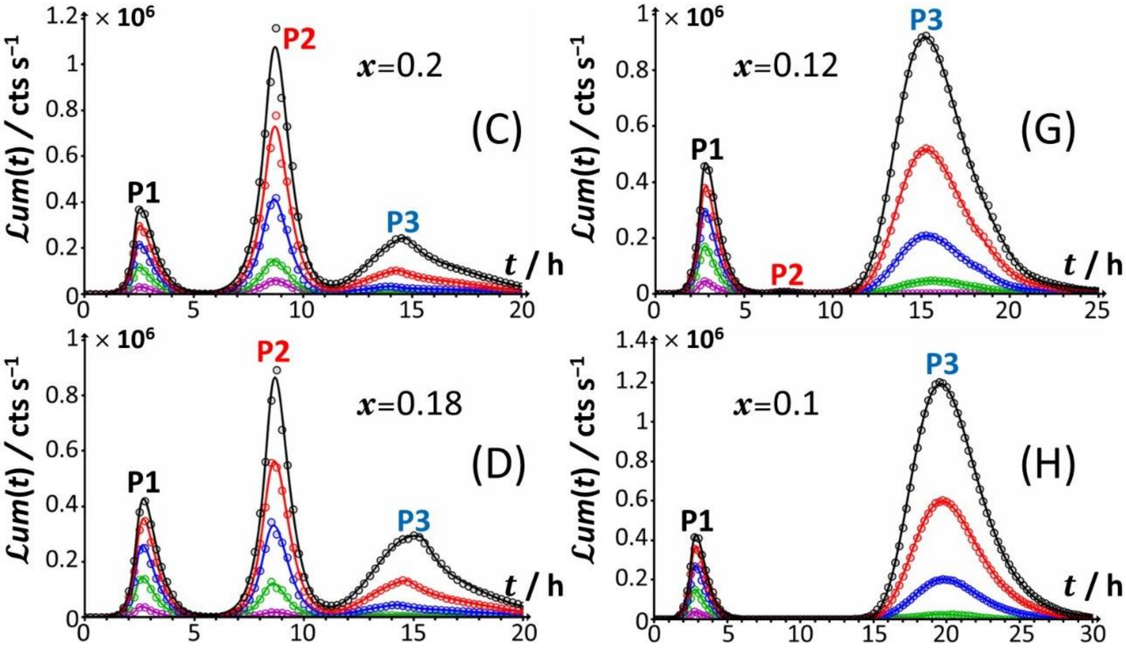

Figure S3. As in **Figure S2** except that the nGGM medium is here supplemented with 0.1% LB and with different concentrations of glucose and xylose as subsumed in the variable *x*, with $0.1 \leq x \leq 1$. Signals are corrected by point-by-point substraction of the corresponding cell response measured in 0 nM Cd concentration.

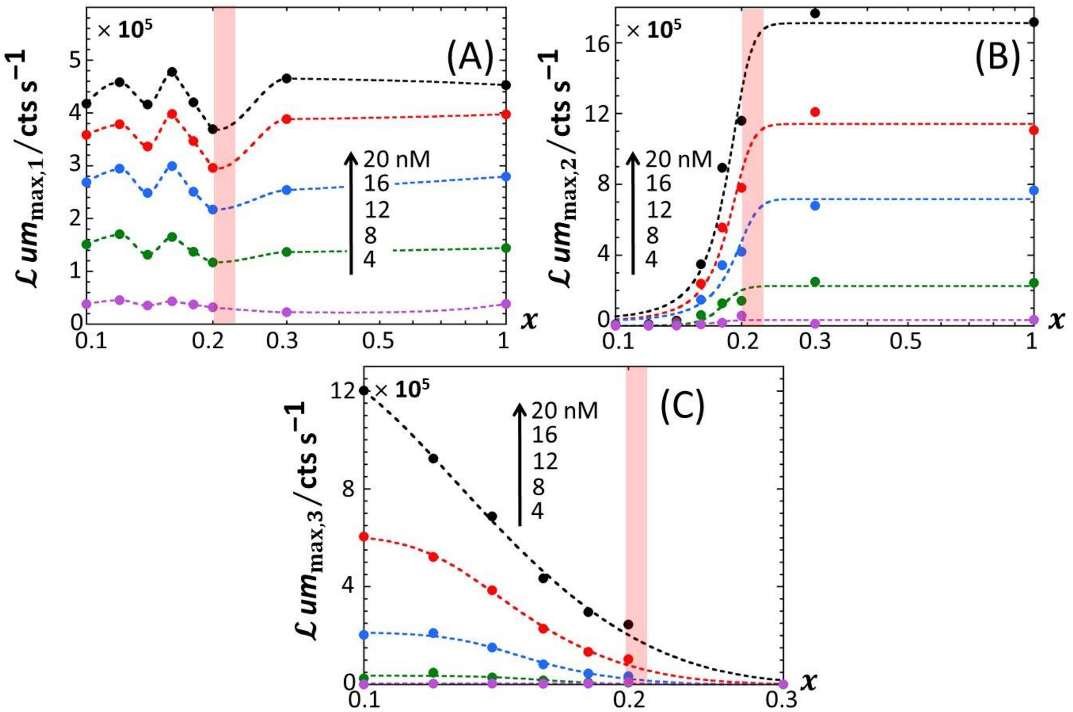

Figure S4. Variations of the maxima of the bioluminescence peaks P1 **(A)**, P2 **(B)** and P3 **(C)** with *x* and total Cd concentration in solution (indicated). The scale in Cd concentration and associated color nomenclature specified in **(A)** apply to panels **(B)** and **(C)**. Symbols : experimental data. Lines: guides to the eyes. The red-colored zone indicates the transition from P1-P2 to P1-P2-P3 signal. Measurement conditions: nGGM media supplemented

with 0.1% LB and different concentrations of glucose and xylose subsumed in the variable *x* with 0.1 ≤ *x* ≤ 1. Data in this figure originate from those given in **Figure S3** (corrected for the reference at 0 nM Cd concentration).

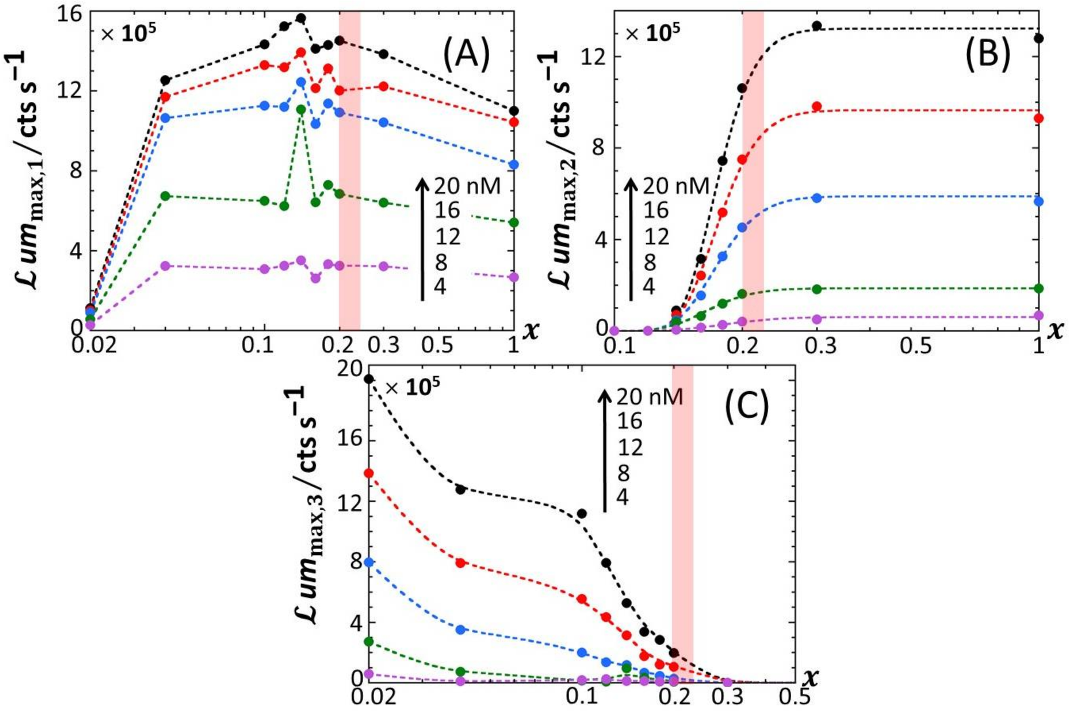

Figure S5. Variations of the maxima of the bioluminescence peaks P1 **(A)**, P2 **(B)** and P3 **(C)** with *x* and total Cd concentration in solution (indicated). The scale in Cd concentration and associated color nomenclature specified in **(A)** apply to panels **(B)** and **(C)**. Symbols : experimental data. Lines: guides to the eyes. The red-colored zone indicates the transition from P1-P2 to P1-P2-P3 signal. Measurement conditions: nGGM media supplemented with 0.15% LB and different concentration ratios *x* in glucose and xylose with 0.02≤ *x* ≤ 1. Data in this figure originate from those given in **Figure S2** (corrected for the reference at 0 nM Cd concentration).

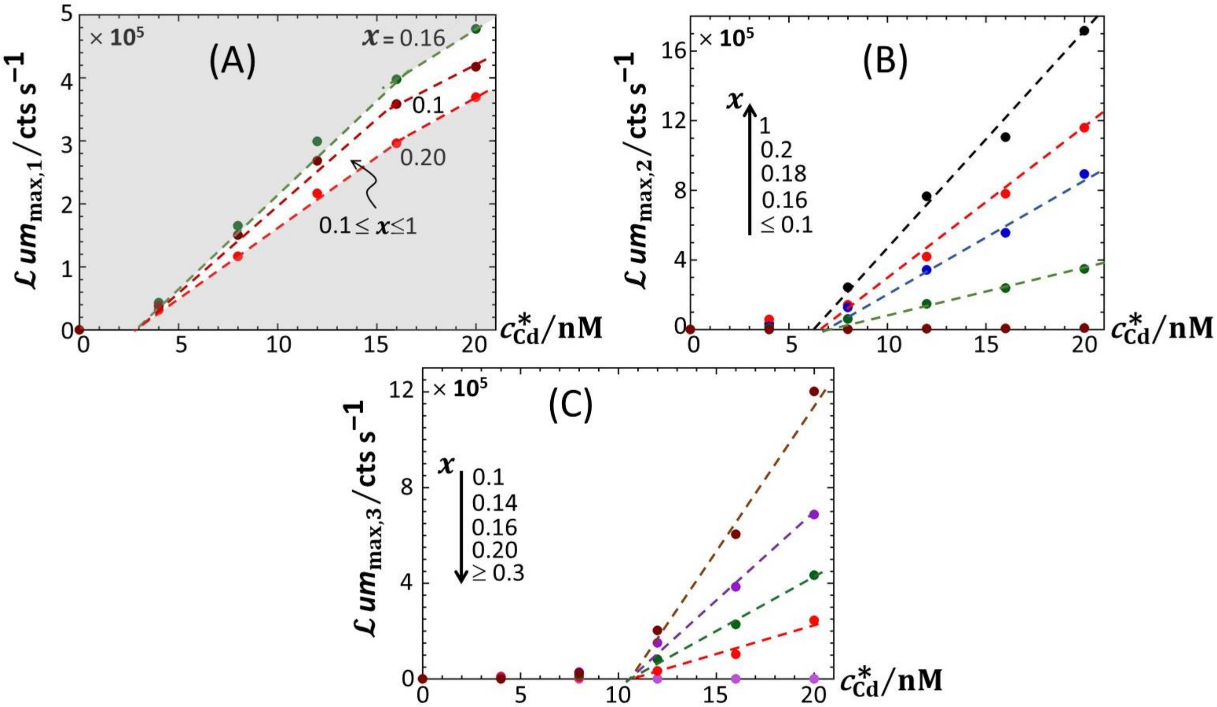

Figure S6. Linear dependence of maxima of the bioluminescence peaks P1 **(A)**, P2 **(B)** and P3 **(C)** on total Cd concentration in solution (indicated) at selected values of *x* (indicated). Symbols : experimental data. Lines: linear regressions. Measurement conditions: nGGM media supplemented with 0.1% LB and different concentration ratios *x* in glucose and xylose with $0.1 \leq x \leq 1$. Data in this figure originate from those given in **Figure S3** (corrected for the reference at 0 nM Cd concentration).

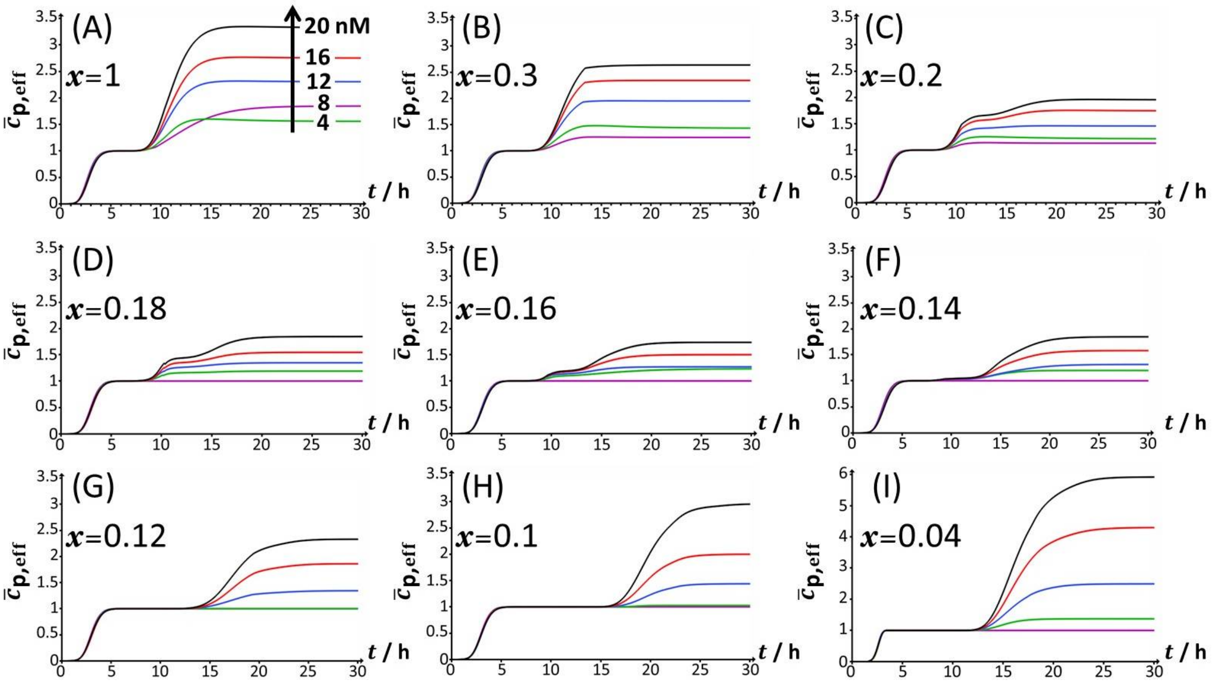

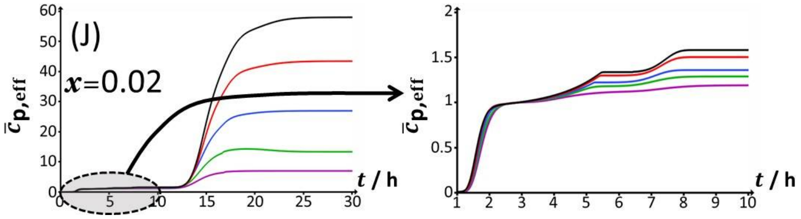

Figure S7. Dependence of the cell photoactivity $\bar{c}_{\mathrm{p,eff}}$ defined by Eq. (10), on time t and total Cd concentration (specified) in 0.15% LB for different glucose to xylose concentration ratios corresponding to $0.02 \leq x \leq 1$ (indicated). The scale in Cd concentration and the associated color nomenclature specified in **(A)** apply to panels **(B)** to **(J)**. $\bar{c}_{\mathrm{p,eff}}(t)$ for given x and Cd concentration conditions is retrieved from theoretical reconstruction of the corresponding measured time-dependent bioluminescence profile displayed in **Figure S2** following the analysis methodology delineated in §3. The theoretical time-dependent bioluminescence patterns corresponding to the $\bar{c}_{\mathrm{p,eff}}(t)$ data given in this **Figure S7** are shown in **Figure S2** (solid lines therein). A zoom for the short-time emission of bioluminescence in panel **(J)** is provided (grey-shaded area).

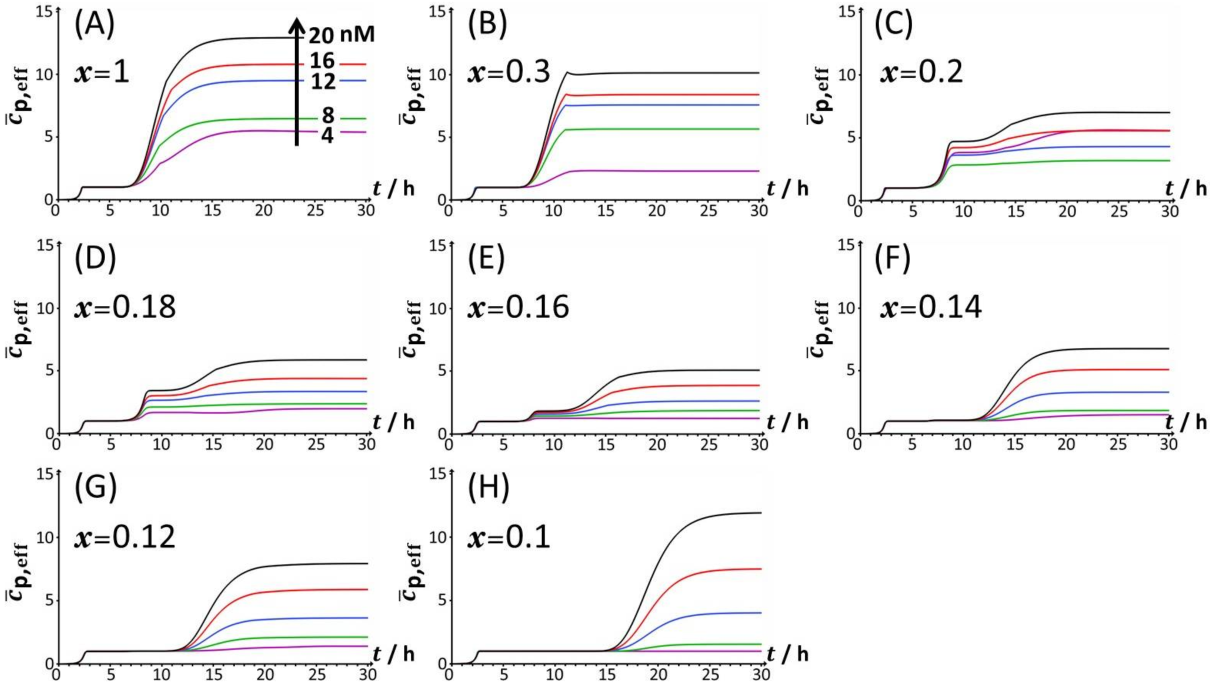

Figure S8. Dependence of the cell photoactivity $\bar{c}_{\mathrm{p,eff}}$ defined by Eq. (10), on time t and total Cd concentration (specified) in 0.10% LB for different glucose to xylose concentration ratios corresponding to $0.1 \leq x \leq 1$ (indicated). The scale in Cd concentration and the associated color nomenclature specified in **(A)** apply to panels **(B)** to **(H).** $\bar{c}_{\mathrm{p,eff}}(t)$ for given x and Cd concentration conditions is retrieved from theoretical reconstruction of the corresponding measured time-dependent bioluminescence profile displayed in **Figure S3** following the analysis methodology delineated in §3. The theoretical time-dependent bioluminescence patterns corresponding to the $\bar{c}_{\mathrm{p,eff}}(t)$ data given in this **Figure S8** are shown in **Figure S3** (solid lines therein).

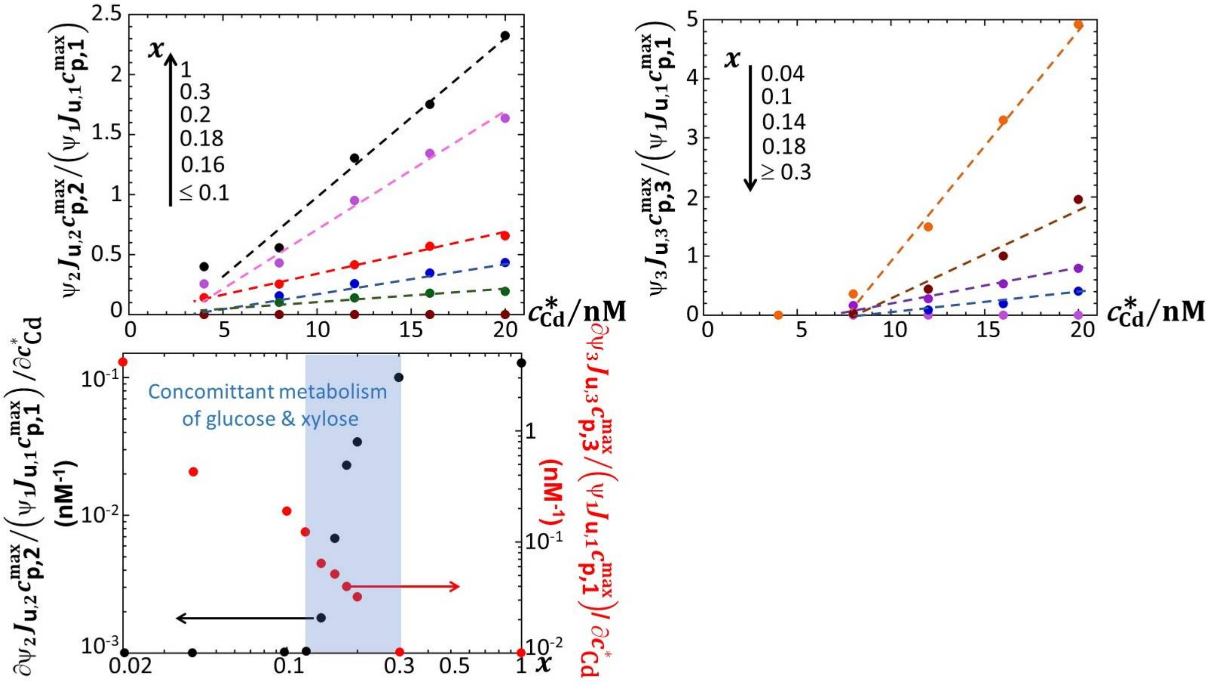

Figure S9. As in **Figure 11** of the main text, albeit for 0.15% LB medium.

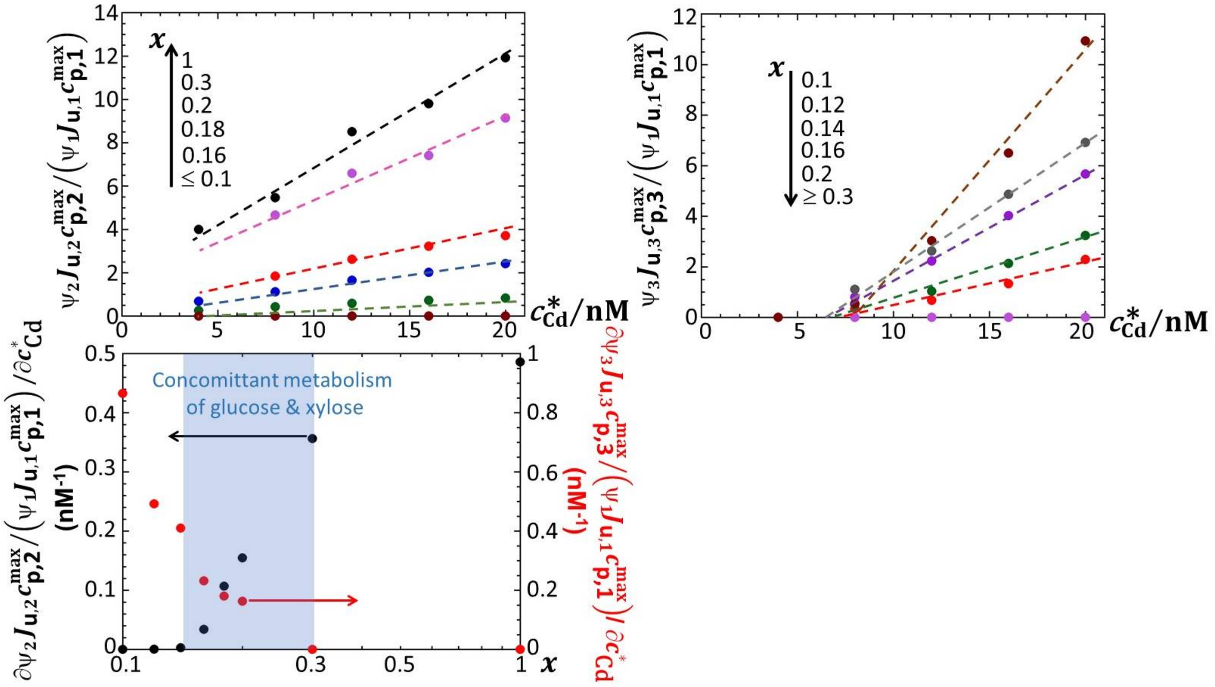

Figure S10. As in **Figure 11** of the main text, albeit for 0.10% LB medium.

Supplementary table (Table S1).

Table S1. Root mean square error (RMSE) values corresponding to the fitting of the normalized bioluminescence signals, collected for Cd-responsive *E. coli* whole-cell biosensor in media supplemented with 0.1% Tryptone, to Eqs. (9)-(10) for the values of total Cd concentration and *x* adopted in this work. The measured signals confronted to theory were those corrected by substraction of 0 nM Cd concentration response and subsequently normalized with respect to the maximum peak amplitude in the first emission mode, as detailed in §3.2 of the main text. The largest RMSE values are obtained at x=0.02 and correspond to the case $\mathcal{L}um_{max,1} << \mathcal{L}um_{max,3}$ with $\mathcal{L}um_{max,1} \rightarrow 0$ (cf. **Figure 2L** in the main text).

[Cd] /nM \ *x*	1	0.5	0.3	0.2	0.18	0.16	0.14	0.12	0.1	0.06	0.04	0.02
4	0.010	0.010	0.009	0.010	0.010	0.011	0.011	0.010	0.008	0.005	0.013	2.75
8	0.008	0.008	0.007	0.023	0.035	0.014	0.024	0.010	0.006	0.006	0.017	0.36
12	0.023	0.015	0.011	0.008	0.012	0.007	0.005	0.006	0.007	0.016	0.029	0.79
16	0.044	0.022	0.018	0.008	0.010	0.009	0.006	0.005	0.009	0.023	0.035	0.66
20	0.020	0.043	0.028	0.014	0.020	0.010	0.008	0.009	0.015	0.036	0.050	0.75
